# Use of low-dose oral theophylline as an adjunct to inhaled corticosteroids in preventing exacerbations of chronic obstructive pulmonary disease: study protocol for a randomised controlled trial

**DOI:** 10.1186/s13063-015-0782-2

**Published:** 2015-06-10

**Authors:** Graham Devereux, Seonaidh Cotton, Peter Barnes, Andrew Briggs, Graham Burns, Rekha Chaudhuri, Henry Chrystyn, Lisa Davies, Anthony De Soyza, Shona Fielding, Simon Gompertz, John Haughney, Amanda J. Lee, Kirsty McCormack, Gladys McPherson, Alyn Morice, John Norrie, Anita Sullivan, Andrew Wilson, David Price

**Affiliations:** Respiratory Medicine, Chest Clinic C, Aberdeen Royal Infirmary, University of Aberdeen, Aberdeen, AB25 2ZN UK; Centre for Healthcare Randomised Trials (CHaRT), University of Aberdeen, Aberdeen, AB25 2ZN UK; Imperial College, National Heart & Lung Institute, Dovehouse Street, London, SW3 6LY UK; Institute of Health & Wellbeing, University of Glasgow, 1 Lilybank Gardens, Glasgow, G12 8RZ UK; Department of Respiratory Medicine, Royal Victoria Infirmary, Newcastle Upon Tyne, NE1 4LP UK; Gartnavel General Hospital, University of Glasgow, Glasgow, G12 0YN UK; Division of Pharmacy & Pharmaceutical Sciences, University of Huddersfield, Huddersfield, HD1 3DH UK; Aintree Chest Centre, University Hospital Aintree, Liverpool, L9 7AL UK; Medical School, University of Newcastle, Newcastle Upon Tyne, NE2 4HH UK; Medical Statistics Team, Division of Applied Health Sciences, University of Aberdeen, Aberdeen, AB25 2ZD UK; Queen Elizabeth Hospital Birmingham, Birmingham, B15 2WB UK; Centre of Academic Primary Care, University of Aberdeen, Aberdeen, AB25 2ZD UK; Cardiovascular and Respiratory Studies, Castle Hill Hospital, Hull, HU16 5JQ UK; Department of Medicine, Norwich Medical School, University of East Anglia, Norwich, NR4 7TJ UK

**Keywords:** COPD, Theophylline, Inhaled corticosteroids, Exacerbation, Randomised controlled trial

## Abstract

**Background:**

Chronic obstructive pulmonary disease (COPD) is associated with high morbidity, mortality, and health-care costs. An incomplete response to the anti-inflammatory effects of inhaled corticosteroids is present in COPD. Preclinical work indicates that ‘low dose’ theophylline improves steroid responsiveness. The Theophylline With Inhaled Corticosteroids (TWICS) trial investigates whether the addition of ‘low dose’ theophylline to inhaled corticosteroids has clinical and cost-effective benefits in COPD.

**Method/Design:**

TWICS is a randomised double-blind placebo-controlled trial conducted in primary and secondary care sites in the UK. The inclusion criteria are the following: an established predominant respiratory diagnosis of COPD (post-bronchodilator forced expiratory volume in first second/forced vital capacity [FEV_1_/FVC] of less than 0.7), age of at least 40 years, smoking history of at least 10 pack-years, current inhaled corticosteroid use, and history of at least two exacerbations requiring treatment with antibiotics or oral corticosteroids in the previous year. A computerised randomisation system will stratify 1424 participants by region and recruitment setting (primary and secondary) and then randomly assign with equal probability to intervention or control arms. Participants will receive either ‘low dose’ theophylline (Uniphyllin MR 200 mg tablets) or placebo for 52 weeks. Dosing is based on pharmacokinetic modelling to achieve a steady-state serum theophylline of 1–5 mg/l. A dose of theophylline MR 200 mg once daily (or placebo once daily) will be taken by participants who do not smoke or participants who smoke but have an ideal body weight (IBW) of not more than 60 kg. A dose of theophylline MR 200 mg twice daily (or placebo twice daily) will be taken by participants who smoke and have an IBW of more than 60 kg. Participants will be reviewed at recruitment and after 6 and 12 months. The primary outcome is the total number of participant-reported COPD exacerbations requiring oral corticosteroids or antibiotics during the 52-week treatment period.

**Discussion:**

The demonstration that ‘low dose’ theophylline increases the efficacy of inhaled corticosteroids in COPD by reducing the incidence of exacerbations is relevant not only to patients and clinicians but also to health-care providers, both in the UK and globally.

**Trial registration:**

Current Controlled Trials ISRCTN27066620 was registered on Sept. 19, 2013, and the first subject was randomly assigned on Feb. 6, 2014.

**Electronic supplementary material:**

The online version of this article (doi:10.1186/s13063-015-0782-2) contains supplementary material, which is available to authorized users.

## Background

Chronic obstructive pulmonary disease (COPD) is a lung disease characterised by progressive airflow obstruction that is not fully reversible and does not change markedly over several months [1]. COPD is common, is caused predominantly by cigarette smoking, and is usually diagnosed from the age of 50 years onwards. In the UK, there arenearly one million diagnosed cases, and COPD accounts for 5–6 % of all deaths (about 28,000 deaths in 2012) [2]. COPD is typically associated with increasing breathlessness on exertion, disability, work absence, premature retirement, morbidity, psychological co-morbidities, reduced quality of life, and premature mortality [1, 3, 4]. COPD is associated with high health-care expenditure: in the UK, National Health Service (NHS) expenditure is about £1 billion per year; for each patient with COPD, the average annual NHS direct costs are £819 (more than £1,300 in severe COPD) [5].

Exacerbations are an important clinical feature of COPD. These are usually precipitated by viral infection or air pollution and are characterised by increasing dyspnoea, cough, sputum expectoration, and malaise. Exacerbations are associated with accelerated lung function decline, reduced physical activity, reduced quality of life, and increased mortality [6–9]. Approximately 15 % of patients with COPD are hospitalised with exacerbations each year. Exacerbations are the second leading cause for emergency hospital admission and account for 60 % of the total direct costs of COPD to the NHS [1, 5]. Typically, 12 % of patients with COPD die in the year following hospitalisation with an exacerbation [9].

Most COPD management guidelines recommend the use of inhaled corticosteroids usually in combination with inhaled long-acting β_2_ agonists to reduce COPD exacerbation rates [1, 10]. However, the responses observed in COPD are not as marked as in asthma; inhaled corticosteroids do not fully suppress airway inflammation in COPD, and patients continue to have exacerbations despite high inhaled corticosteroid doses [11]. Although inhaled corticosteroids are beneficial in COPD, the airway inflammation in COPD is relatively insensitive to their anti-inflammatory effects even at high doses [12–14].

Oral theophylline has been used conventionally as a bronchodilator in COPD for over 70 years; however, to achieve modest clinical effects, relatively high blood levels (10–20 mg/l) with clinical monitoring are required. Unfortunately, the therapeutic index of ‘high dose’ theophylline is narrow. Prior use at ‘high dose’ as a bronchodilator frequently resulted in drug concentrations close to those where side effects were encountered, namely nausea, gastrointestinal upset, cardiac arrhythmias, and malaise. Not surprisingly, use of ‘high dose’ theophylline has declined in recent years and is being replaced by better-tolerated inhaled bronchodilator therapies. Recently, however, preclinical studies have demonstrated that theophylline at ‘low dose’ (plasma concentration of 1–5 mg/l) increases the sensitivity of COPD airway inflammation to the anti-inflammatory effects of inhaled corticosteroids [15–22]. The concept that ‘low dose’ theophylline may produce a beneficial synergistic effect by increasing the corticosteroid sensitivity in COPD is supported by two small randomised controlled trials with inflammatory indices as primary outcomes and a Canadian health administration database study [23–25]. The potential benefit of using ‘low dose’ theophylline to increase corticosteroid responsiveness in COPD is that, when used in combination with inhaled corticosteroids, it should reduce exacerbation rates. Moreover, ‘low dose’ theophylline is inexpensive and is anticipated to avoid the side effects encountered with conventional ‘high dose’ theophylline, making blood monitoring unnecessary.

Here, we describe the Theophylline With Inhaled Corticosteroids (TWICS) study, a randomised double-blind placebo-controlled trial that will test the hypothesis that in patients with COPD established on a treatment regimen including an inhaled corticosteroid, the addition of oral ‘low dose’ theophylline will reduce the rate of exacerbation. The full protocol is available as Additional file 1.

## Methods/Design

### Trial design

**Fig. 1:**
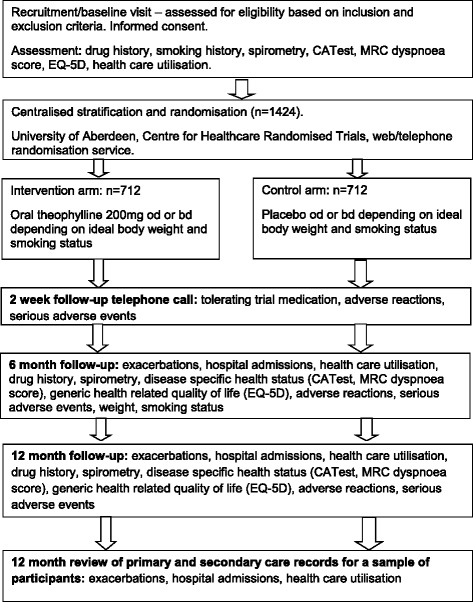
Flow diagram of study design and schedule. bd, Twice a day (*Bis in die*); CATest, COPD (Chronic Obstructive Pulmonary Disease) Assessment Test; EQ-5D, EuroQOL five-dimension questionnaire; MRC, Medical Research Council; od, Once a day (*Omne in die*)

TWICS is a double-blind randomised, placebo-controlled, UK multicentre clinical trial comparing the addition of ‘low dose’ theophylline or placebo for 52 weeks with current COPD therapy that includes inhaled corticosteroids in COPD patients who in the previous year have had two or more exacerbations of COPD treated with oral corticosteroids or antibiotics. Fig. 1 provides a schematic representation of study design and schedule.

The trial is set in primary and secondary care sites in the UK. In primary care, some general practices act as recruitment sites, whereas others act as Participant Identification Centres with identified participants being evaluated in other primary or secondary care recruitment sites. The first subject was randomly assigned on Feb. 6, 2014. In total, 1424 eligible people with COPD will be randomly assigned to receive visually identical ‘low dose’ theophylline (Uniphyllin MR 200 mg once or twice daily) or placebo for 52 weeks. The trial is approved by Scotland A Research Ethics Committee (#13/SS/0081, June 28, 2013) and the Medicines and Healthcare Products Regulatory Agency (EudraCT 2013-001490-25, CTA 21583/0218/001, Aug. 8, 2013). All participants are required to give written informed consent.

### Participants

Inclusion criteria comprise an established predominant respiratory diagnosis of COPD (Global Initiative on Obstructive Lung Disease [GOLD]/National Institute for Health and Care Excellence [NICE] Guideline definition: post-bronchodilator forced expiratory volume in first second/forced vital capacity [FEV_1_/FVC] of less than 0.7), age of at least 40 years, a smoking history of at least 10 pack-years, current use of inhaled corticosteroid therapy, a history of at least two exacerbations requiring treatment with antibiotics or oral corticosteroids in the previous year (based on patient report). In addition, participants must be clinically stable at the time of recruitment (no COPD exacerbation for at least 4 weeks).

The main exclusion criteria are current use of theophylline, hypersensitivity to theophylline, or use of drugs known to interact with theophylline or increase serum theophylline [26] (for full list, see Additional file 1). Further exclusion criteria are a predominant respiratory disease other than COPD, unstable ischaemic heart disease, or any other clinically important disease/disorder which, in the investigator’s opinion, either puts the participant at risk because of study participation or may influence the results of the study or the participant’s ability to take part in the study. For women, current pregnancy or breast-feeding and planned pregnancy during the study are exclusion criteria.

### Identification, recruitment, and randomisation

Potential participants are recruited from both primary and secondary care across the UK with the aim of recruiting the majority of participants (more than 50 %) from primary care. Recruitment strategies differ between centres, depending on local geographic and NHS organisational factors.

In primary care, potential participants are identified from searches of general-practice databases. In some centres, COPD community matrons and other community-based intermediate care services for patients with COPD are available and are used to identify potential participants. In secondary care, potential participants are identified from those patients who are attending hospital respiratory out-patient clinics, spirometry services, or smoking cessation services. Some trial centres also have access to volunteer databases/registries. Potential participants are sent an invitation letter and a patient information leaflet informing them of the trial aims and level of involvement required. The letter provides a variety of methods for interested potential participants to contact the local trial team.

At the recruitment/baseline visit at the local trial centre, eligibility is established, the trial explained, questions addressed, and informed written consent provided by the participant.

Participants are randomly assigned by using a computerised randomisation system available as both an interactive voice response telephone system and an internet-based application. The randomisation service is administered by the Centre for Healthcare Randomised Trials (CHaRT), University of Aberdeen. Consenting patients will be stratified by region of trial centre and recruitment setting (primary and secondary) and then randomly assigned with equal probability to the intervention and control arms.

### Intervention

The active intervention is Uniphyllin MR 200 mg tablets and a visually identical control placebo. The packaging and labelling of active and placebo interventions are identical. Intervention is for 52 weeks of therapy.

‘Low dose’ theophylline dosing is based on pharmacokinetic modelling of theophylline and incorporates the major determinants of theophylline steady-state concentration (i.e., weight, smoking status, and theophylline clearance [27, 28]) and is designed to achieve a steady-state serum theophylline level of 1–5 mg/l and certainly less than 10 mg/l (more than 10 mg is the level associated with ‘high dose’ theophylline, possible side effects, and augmentation of corticosteroid insensitivity) (Appendix 1 of the Additional file 1). Dosing is determined by the participant’s ideal body weight (IBW) and self-reported smoking status. IBW is computed by using the Devine formulae: IBW_female_ = 45 + 0.9 (height in cm – 152) kg, and IBW_male_ = 50 + 0.9 (height in cm – 152) kg [29].

A dose of theophylline MR 200 mg once daily (one placebo once daily) is taken by participants who do not smoke or participants who smoke but have an IBW of not more than 60 kg. A dose of theophylline MR 200 mg twice daily (one placebo twice daily) is taken by participants who smoke and have an IBW of more than 60 kg.

To be classed as a “non-smoker”, a participant must have abstained from smoking for at least 12 weeks. If less than the IBW, the actual body weight is used to determine dose. When informed of their patient’s participation in the trial, general practitioners are advised to manage their patient for exacerbations as in normal clinical practice but to assume that the participant is taking low-dose theophylline, and the prescription of interacting drugs that increase serum theophylline levels should be avoided. In the event that drugs that interact to increase theophylline concentration have to be prescribed for 3 weeks or less, patients are asked to suspend taking study medication and recommence their study medication after the course of interacting drug has been completed. If the interacting drug is prescribed for more than 3 weeks, participants discontinue the study medication but remain in the study and are followed up in accordance with the trial protocol.

In secondary care trial sites, the first pack of 4-week study medication is dispensed from the local Clinical Trials Pharmacy. In primary care trial sites, the first pack of study medication is couriered to the participant’s home from the lead Clinical Trials Pharmacy in Aberdeen. Subsequent packs containing a 24-week drug supply are delivered to the participants’ homes at around weeks 3 and 27 via a signed-for delivery service operated by a third party. Receipt of trial medication is confirmed by signature on delivery. Written informed consent to pass on a participant’s name and address to the third-party distributer is obtained at recruitment.

### Primary outcome variables

The primary outcome measure is the total number of exacerbations of COPD necessitating changes in management (minimum management change—use of oral corticosteroids or antibiotics) during the 52-week treatment period, as reported by the participant. The primary economic outcome measure is cost per quality-adjusted life year (QALY) gained during the 52-week treatment period.

### Secondary outcome variables

During the 52-week treatment period, the secondary outcomes are the following: number of participant-reported COPD exacerbations requiring hospital admission, number of episodes of pneumonia, number of emergency hospital admissions (all causes), post-bronchodilator lung function (FEV_1_FVC), all-cause and respiratory mortality, serious adverse events, adverse reactions, total dose of inhaled corticosteroid, utilisation of primary or secondary health care for respiratory events, disease-specific health status (COPD Assessment Test, or CAT) [30], Medical Research Council (MRC) dyspnoea scale [31], generic health-related quality of life (EQ-5D) [32], and modelled lifetime incremental cost per QALY.

### Follow-up and data collection

Participants are reviewed at face-to-face assessments at recruitment and after 6 and 12 months of treatment. Approximately 2 weeks after enrolment, participants are contacted by telephone to ascertain whether they are tolerating the study intervention.

In the event that a participant is unable to attend a scheduled follow-up assessment visit because of an acute illness (e.g., exacerbation of COPD) or other reasons, the visit can be postponed, ideally within 4 weeks of the scheduled assessment visit. Participants unable to attend a face-to-face assessment at 6 and 12 months are followed up by telephone or a home visit or sent the questionnaire to complete at home. The schedule for data collection within the study is outlined in Table 1.

**Table 1:** Schedule of study assessments

Assessment	Recruitment	2 weeks (phone)	Month 6 (face to face)	Month 12 (face to face)	Post-study GP records
Assessment of eligibility criteria	X				
Written informed consent	X				
Clinical history	X				
Drug history	X		X	X	
Smoking status	X	X	X	X	
Height	X				
Weight	X		X	X	
Total number COPD exacerbations requiring oral corticosteroids/antibiotics			X	X	X
Hospital admissions			X	X	X
Health-related quality of life (EQ-5D)	X		X	X	
Disease-related health status (CAT, MRC dyspnoea)	X		X	X	
Post-bronchodilator lung function	X		X	X	
Adverse events/drug reactions		X	X	X	
Health-care utilisation			X	X	
Patient compliance			X	X	

X boxes represent which aspect of the assessment is conducted. *CAT* COPD (Chronic Obstructive Pulmonary Disease) Assessment Test, *COPD* Chronic obstructive pulmonary disease, *EQ-5D* EuroQOL five-dimension questionnaire, *GP* General practitioner, *MRC* Medical Research Council

The following data are collected:

#### Drug history

Regular use of prescription drugs is recorded at recruitment as free text and at the 6- and 12-month assessments.

#### Smoking history

Smoking history (age commenced, age ceased, and average cigarettes smoked per day) is recorded at recruitment and at the 6- and 12-month assessments. Pack-year consumption is computed at recruitment.

#### Height and weight

Height and weight are measured at recruitment, and weight is measured at the 6- and 12-month assessments.

#### Number of chronic obstructive pulmonary disease exacerbations

The primary outcome measure of the total number of COPD exacerbations requiring antibiotics/oral corticosteroids whilst on study medication is ascertained at the 6- and 12-month assessments. The total number of participant-reported COPD exacerbations will be validated for approximately 20 % of randomly identified participants by examination of primary care records after the study. Concordance between participant-recorded and primary care records will be computed by using percentage agreement, and more than 80 % will be deemed acceptable [33].

The American Thoracic Society/European Respiratory Society guideline definition of COPD exacerbation is used: a worsening of patient’s dyspnoea, cough, or sputum beyond day-to-day variability sufficient to warrant a change in management [34]. The minimum management change will be treatment with antibiotics or oral corticosteroids. A minimum of 2 weeks between consecutive hospitalisations/start of new therapy is necessary to consider events as separate. A modified American Thoracic Society/European Respiratory Society operational classification of exacerbation severity will be used for each exacerbation: level I, increased use of their short-acting β_2_ agonist; level II, use of oral corticosteroids or antibiotics; level III, care by services to prevent hospitalisation; level IV, admitted to hospital [34].

#### Hospital admissions

The number of emergency hospital admissions whilst on study medication is ascertained at the 6- and 12-month assessments. COPD-associated emergency admissions are also identified. The number of participant-reported hospital admissions will be validated for a randomly identified sample of 20 % of participants by examination of primary and secondary care records after the study. Concordance between participant-recorded and primary/secondary care records will be computed by using percentage agreement, and more than 80 % will be deemed acceptable [33].

#### Health-related quality of life

Health-related quality of life data are captured at recruitment and at the 6- and 12-month assessments by questionnaire using the EuroQoL 5D (EQ-5D 3-level version) Index [32], which has been used widely in COPD. The completed instrument can be translated into quality-of-life utilities suitable for calculation of QALYs through the published UK tariffs [35].

#### Disease-related health status

Disease-related health status is ascertained at recruitment and at the 6- and 12-month assessments by questionnaire using the CAT [30]. The CAT is an eight-item unidimensional measure of health status impairment in COPD and is completed by the subject. The CAT has a scoring interval of 0–40; 0–5 is the norm for healthy non-smokers, and more than 30 is indicative of a very high impact of COPD on quality of life [30]. The CAT is reliable and responsive, correlates very closely with the St George Respiratory Questionnaire, and is preferred because it provides a more comprehensive assessment of the symptomatic impact of COPD [30, 36, 37].

The MRC dyspnoea scale is included in the recruitment and the 6- and 12-month assessments [31]. The MRC dyspnoea scale has been in use for many years to grade the effect of breathlessness on daily activities. The MRC dyspnoea scale is a single question which assesses breathlessness related to activities. The scoring interval is 1–5; 1 refers to ‘Not troubled by breathlessness except on strenuous exercise’, and 5 indicates ‘Too breathless to leave the house or breathless when dressing or undressing’. The MRC score has been validated against walking test performance and other metrics of COPD health status (e.g., St George Respiratory Questionnaire [38]).

#### Post-bronchodilator lung function

Post-bronchodilator lung function is measured at recruitment and 6 and 12 months by using spirometry performed to American Thoracic Society/European Respiratory Society standards [39].

#### Health-care utilisation

In keeping with the NHS perspective adopted for the economic analysis, health-care utilisation is the focus of the costing for the study. This includes the study drug, concomitant medications, general practitioner visits, and any-cause hospitalisations during the previous 6 months and is ascertained at the 6- and 12-month assessments.

#### Adverse reactions and serious adverse events

The trial complies with the UK NHS National Research Ethics Service guidelines for reporting adverse events [40]. Adverse reactions and serious adverse events whilst on study medication are ascertained at the 2-week telephone call and the 6- and 12-month assessments. Participants are notified of recognised adverse reactions and encouraged to contact the local study centre if they experience these.

#### Mortality

Deaths during the follow-up period are recorded and are reported as serious adverse events.

#### Compliance

Compliance with study medication is assessed at the 6- and 12-month assessments. Participants will be asked to return empty drug bottles and unused medication; compliance will be calculated by pill counting [41].

### Sample size

The sample size of 1424 was estimated on the basis of the ECLIPSE (Evaluation of COPD Longitudinally to Identify Predictive Surrogate Endpoints) study reporting the frequency of COPD exacerbation in 2138 patients [42]. For patients identical to our target population (who in a 1-year period have at least two self-reported COPD exacerbations requiring antibiotics or oral corticosteroids), the mean (standard deviation) number of COPD exacerbations within 1 year was 2.22 (1.86) [42]. Given a similar rate in the placebo arm, 669 subjects are needed in each arm of the trial to detect a clinically important reduction in COPD exacerbations of 15 % (i.e., from a mean of 2.22 to 1.89) with 90 % power at the two-sided 5 % significance level. With an estimated 6 % loss to follow-up, 712 participants are required in each study arm (i.e., 1424 in total).

### Statistical methods

All analyses will be governed by a comprehensive statistical analysis plan that is in place and will be in accordance with the intention-to-treat principle with a per-protocol analysis performed as a sensitivity. The per-protocol analysis will exclude participants who were not compliant (at less than 75 %) with their study medication.

#### Primary clinical outcome

The number of COPD exacerbations requiring antibiotics or oral corticosteroids in the 52-week treatment period will be compared between randomised groups by using a generalised linear model with log-link function, an appropriate dispersion parameter, and length of time in the study as an offset. Estimates will be adjusted for centre and other baseline covariates that are known to be strongly related to outcome (e.g., age, smoking, and COPD hospitalisations in the year prior to study). An over-dispersion parameter will be used to adjust for between-patient variability.

#### Economic evaluation

An NHS perspective will be adopted in keeping with the NICE reference case for health technology assessments [43]. The health economic evaluation will be conducted in two stages. First, the cost-effectiveness of treatment will be calculated for the within-trial period on the basis of observed data. Second, the results of the trial will be extrapolated to patient lifetimes by using cost-effectiveness modelling.

The within-trial analysis will make use of the health-care resource use data (translated to a cost per patient by using unit cost standard reference sources), the exacerbation rate associated with the treatment arms, and the quality-of-life effects estimated from the EQ-5D combined with utility data to calculate QALYs. Non-parametric bootstrapping will be used to capture sampling uncertainty in the observed data, and results will be presented as cost per exacerbation avoided and cost per QALY gained within the trial period with accompanying confidence intervals (or cost-effectiveness acceptability curves if more appropriate). The extrapolation analysis will make use of regression estimates of exacerbation on cost and quality of life from the trial, as well as previously published models of COPD, to guide the extrapolation to patient lifetimes. In addition to sampling uncertainty, extensive sensitivity analysis will be performed to understand the importance of alternative modelling assumptions for the extrapolated results.

## Discussion

COPD is a common disease associated with high morbidity, mortality, and health-care costs despite the widespread use of inhaled corticosteroids. Although inhaled corticosteroids are beneficial in COPD, a relative insensitivity of COPD airway inflammation to the anti-inflammatory effects of high-dose inhaled corticosteroids has been demonstrated [12–14]. TWICS is a randomised double-blind placebo-controlled trial that tests the hypothesis: Does the addition of oral ‘low dose’ theophylline reduce the rate of exacerbation in patients with COPD established on a treatment regimen including an inhaled corticosteroid?

The primary outcome of COPD exacerbations is clinically important for patients, their carers, and health services; exacerbations of COPD are associated with many adverse outcomes, including mortality, and their management comprises 60 % of NHS expenditure for COPD [5]. To be eligible in TWICS, participants must have an established diagnosis of COPD on the basis of the spirometric finding of FEV_1_/FVC of less than 0.7 and of at least two exacerbations in the previous year. These criteria reflect the findings of the ECLIPSE study that the single best predictor of exacerbations is a history of exacerbations [42]. Moreover, patients of the frequent-exacerbation phenotype (of at least two exacerbations in a year) are present at all severities of COPD (22 % of GOLD stage 2, 33 % of stage 3, and 47 % of stage 4), and the frequent-exacerbation phenotype is relatively stable over a 3-year period and can be identified on the basis of patient recall.

It is almost certain that a substantial proportion of the participants in TWICS will have severe lung disease and will have limited exercise tolerance. Allowances have been made in the trial design to facilitate participation by this group of patients: at site discretion, participants can be recruited at home, and those unable to attend follow-up assessment visits will be assessed by telephone review and postal collection of quality-of-life questionnaires; the majority (at least 48 of 52 weeks) of study medication will be couriered directly to the homes of participants, thus avoiding travel to study centres to collect supplies.

Oral theophylline has conventionally been used primarily as a bronchodilator in COPD for over 70 years; however, to achieve modest clinical effects, relatively high blood levels (10–20 mg/l) are required. The bronchodilator effect of this ‘high dose’ theophylline is the consequence of inhibition of phosphodiesterase and consequent relaxation of airway smooth muscle; however, phosphodiesterase inhibition is also associated with the side effects of theophylline, namely nausea, gastrointestinal upset, cardiac arrhythmias, and malaise. The use of high-dose theophylline has declined in recent years, and current COPD guidelines have relegated high-dose theophylline to third-line therapy because of its narrow therapeutic index, modest clinical effect, side effect profile, drug interactions, the need for monitoring and the development of inhaled long-acting β_2_ agonists, anti-muscarinics, and the widespread use of inhaled corticosteroids [1, 10]. The use of ‘low dose’ theophylline derives from the demonstration by preclinical studies and two small randomised controlled trials that theophylline at ‘low dose’ (plasma concentration of 1–5 mg/l) increases the sensitivity of COPD airway inflammation to the anti-inflammatory effects of inhaled corticosteroids [15–22]. Previous studies have investigated the potential anti-inflammatory effects of ‘low dose’ theophylline in COPD and asthma (not in conjunction with inhaled corticosteroids). However, they have used a ‘one size fits all’ dosing approach (e.g., all participants received 100 mg twice daily or 200 mg twice daily) [20, 23, 24, 44–46]. In contrast, in TWICS, theophylline dosing is stratified, as determined by IBW and smoking status. Population studies have demonstrated that theophylline pharmacokinetics are influenced by weight, COPD disease status (reduced clearance), and smoking (increased clearance) [28, 47–56]. Smoking induces theophylline clearance by approximately 60 %, which gradually returns to normal levels upon smoking cessation, and this has been incorporated into the definition of a non-smoker in TWICS. The use of IBW in preference to actual weight avoids the potential for giving an inappropriately high dose of theophylline to obese participants; furthermore, use of IBW is good clinical practice. In TWICS, theophylline dosing is based on pharmacokinetic modelling incorporating the major determinants of theophylline steady-state concentration, i.e., weight, smoking status, and clearance of theophylline (low, normal, or high), and is designed to achieve a steady-state serum theophylline level of 1–5 mg/l and certainly less than 10 mg/l. Theophylline is metabolised in the liver by the enzyme CYP1A2, which is induced by smoking and inhibited by a number of medications with a consequent increase in serum theophylline levels [26]. For this reason, the exclusion criteria include long-term use of drugs known to increase serum theophylline.

Theophylline in the form of intravenous aminophylline has been used in the treatment of severe acute exacerbations of COPD in hospital settings. However, research does not support this modality of treatment and this is reflected in guideline recommendations [1, 10], and the use of intravenous aminophylline has rapidly declined. When used, intravenous aminophylline is usually administered as a loading dose followed by a maintenance infusion in patients not established on theophylline, and for patients established on theophylline, only the maintenance infusion is given because of toxicity concerns. Inevitably, during TWICS, some participants will be admitted to hospital with very severe life-threatening exacerbations of COPD, and the attending physician may wish to use intravenous aminophylline. Pharmacokinetic modelling demonstrates that patients receiving ‘low dose’ theophylline will not achieve toxic levels of theophylline following the usual loading dose of aminophylline, because their baseline serum theophylline concentrations will vary between 1 and 5 mg/l; and after the loading dose of aminophylline, serum theophylline will remain within the conventional bronchodilating interval of 10–20 mg/l. This is clinically important as the attending physician will not be aware whether a TWICS participant is on theophylline or placebo, and the modelling confirms that an aminophylline infusion can be safely administered if thought by the attending physician to be clinically indicated. Study medication will be suspended whilst the participant receives intravenous aminophylline and restarted when the aminophylline discontinued. In keeping with guideline recommendations, serum theophylline will be measured 24 h after commencing intravenous aminophylline (allocation status will not be discernible from such a level) [1, 10]. All participants are given (and advised to carry) a credit card-sized alert card giving brief information about the trial with advice for clinicians, contact details for the local investigator, and the contact details for emergency unblinding. The participant’s primary care physician is informed of participation and provided with appropriate clinical advice.

Theophylline has been used for decades, and many clinicians are familiar with its use; moreover, ‘low dose’ theophylline is considerably less expensive than inhaled therapies and does not incur the costs of monitoring of blood levels. The demonstration that low-dose theophylline increases the efficacy of inhaled corticosteroids in COPD by reducing the incidence of exacerbations will be relevant not only to patients and clinicians but also to health-care providers, both in the UK and globally.

## Trial status

The first subject was recruited on February 6, 2014, and the trial is currently recruiting patients. The anticipated date of last participant assessment is October 2016.

## Additional file

Additional file 1:
**Title: TWICS study protocol. Description: Full TWICS study protocol.**

## Abbreviations

CAT: COPD (Chronic Obstructive Pulmonary Disease) Assessment Test
COPD: Chronic obstructive pulmonary disease
ECLIPSE: Evaluation of COPD Longitudinally to Identify Predictive Surrogate Endpoints
EQ-5D: EuroQOL five-dimension questionnaire
FEV_1_: Forced expiratory volume in first second
FVC: Forced vital capacity
GOLD: Global Initiative on Obstructive Lung Disease
IBW: Ideal body weight
MR: modified release
MRC: Medical Research Council
NHS: National Health Service
NICE: National Institute for Health and Care Excellence
QALY: Quality-adjusted life year
TWICS: Theophylline With Inhaled Corticosteroids
