# Supplementary material for: Use of low-dose oral theophylline as an adjunct to inhaled corticosteroids in preventing exacerbations of chronic obstructive pulmonary disease: study protocol for a randomised controlled trial
Source: Trials. 2015 Jun 10;16:267. doi: 10.1186/s13063-015-0782-2 (PMC4465017; doi:10.1186/s13063-015-0782-2)
Supplement: Additional file 1: — Title: TWICS study protocol. Description: Full TWICS study protocol. [file 13063_2015_782_MOESM1_ESM.pdf]

Study Protocol

A randomised, double-blind placebo controlled trial of the effectiveness of low dose oral theophylline as an adjunct to inhaled corticosteroids in preventing exacerbations of chronic obstructive pulmonary disease

TWICS (Theophylline With Inhaled Corticosteroid)

Sponsor	University of Aberdeen / NHS Grampian
Funder	NIHR Health Technology Assessment
Funding Reference Number	11/58/15
Co-Chief Investigators	Graham Devereux, David Price
Sponsor Reference Number	3/016/13
EudraCT Number	2013-001490-25
CTA Number	21583/0218/001
REC Number	13/SS/0081
ISRCTN Number	27066620
Version Number and Date	Version 6, 4 August 2014

TRIAL / STUDY PERSONNEL AND CONTACT DETAILS

Sponsor:	University of Aberdeen,<br>Dr Gail Holland,<br>Research Governance Manager,<br>Polwarth Building, Foresterhill,<br>Aberdeen.<br>AB25 2ZD<br>Telephone: 01224437073<br>E-mail: g.holland@abdn.ac.uk
Co-Chief investigators:	Prof Graham Devereux,<br>Professor of Respiratory Medicine,<br>Chest Clinic C,<br>Aberdeen Royal Infirmary,<br>Foresterhill, Aberdeen. AB25 2ZN<br>Telephone: 01224 438476<br>E-mail: g.devereux@abdn.ac.uk<br><br>Prof David Price,<br>Professor of Primary Care Respiratory Medicine,<br>Respiratory Medicine, Primary Care,<br>Centre of Academic Primary Care,<br>West Block, Polwarth Building,<br>Foresterhill,<br>Aberdeen. AB25 2ZD<br>Telephone: 02081233923<br>E-mail: david@respiratoryresearch.org)
Co-investigators:	Prof Peter Barnes,<br>Head of Respiratory Medicine,<br>National Heart & Lung Institute,<br>Dovehouse St, London. SW3 6LY<br>Telephone: 0207 351 8174<br>E-mail: p.j.barnes@imperial.ac.uk<br><br>Prof Andy Briggs,<br>Chair in Health Policy & Economic Evaluation,<br>Health Economics & Health Technology Assessment,<br>Centre for Population & Health Sciences,<br>1 Lilybank Gardens, Glasgow. G12 8RZ<br>Telephone: 0141 330 5017<br>E-mail: Andrew.Briggs@glasgow.ac.uk<br><br>Dr Rekha Chaudhuri,<br>Associate Professor and Associate Specialist,<br>University of Glasgow<br>Gartnavel General Hospital,<br>1053 Great Western Road, Glasgow. G12 0YN<br>Telephone: 0141 211 1673<br>E-mail: rekhachaudhuri@yahoo.com

Co-investigators *(cont)*:	Prof Henry Chrystyn,<br>Head of Pharmacy,<br>Division of Pharmacy & Pharmaceutical Sciences,<br>University of Huddersfield,<br>Huddersfield. HD1 3DH<br>Telephone: 01484 472783<br>E-mail: h.chrystyn@hud.ac.uk<br><br>Dr Seonaidh Cotton,<br>Trials Manager,<br>3rd Floor, Health Sciences Building,<br>Foresterhill, Aberdeen. AB25 2ZD<br>Telephone: 01224 438178<br>E-mail: s.c.cotton@abdn.ac.uk<br><br>Dr Lisa Davies,<br>Consultant Chest Physician,<br>Aintree Chest Centre,<br>University Hospital Aintree,<br>Lower Lane, Liverpool, L9 7AL<br>Telephone: 0151 529 3796<br>E-mail: lisa.davies@aintree.nhs.uk<br><br>Dr Anthony De Soyza,<br>HEFCE Academic Clinical Senior Lecturer,<br>Medical School, Framlington Place,<br>Newcastle Upon Tyne. NE2 4HH<br>Telephone: 0191 213 7468<br>E-mail: anthony.de-soyza@newcastle.ac.uk<br><br>Dr John Haughney,<br>Research Fellow,<br>Centre of Academic Primary Care,<br>West Block, Polwarth Building, Foresterhill,<br>Aberdeen. AB25 2ZD<br>Telephone: 01224 553066<br>E-mail: john.haughney@btinternet.com<br><br>Prof Alyn Morice,<br>Head of Cardiovascular and Respiratory Studies,<br>Castle Hill Hospital, Hull. HU16 5JQ<br>Telephone: 01482 624066<br>E-mail: A.H.Morice@hull.ac.uk<br><br>Prof John Norrie,<br>Director, CHaRT,<br>3rd Floor, HSRU, Health Sciences Building,<br>Foresterhill, Aberdeen. AB25 2ZD<br>Telephone: 01224 438149<br>E-mail: j.norrie@abdn.ac.uk

Co-investigators *(cont)*:	Dr Anita Sullivan,<br>Consultant Respiratory Physician,<br>7th Floor Area 2, New Queen Elizabeth Hospital,<br>Birmingham, Mindelsohn Way, Edgbaston,<br>Birmingham. B15 2WB<br>Telephone: 07798 842561<br>E-mail: Anita.Sullivan@uhb.nhs.uk<br><br>Dr Andrew Wilson,<br>Clinical Senior Lecturer,<br>Airway Inflammation Research Group,<br>Biomedical Research Centre,<br>School of Medicine, University of East Anglia,<br>Norwich. NR4 7TJ<br>Telephone: 01603 591257<br>E-mail: A.M.Wilson@uea.ac.uk<br><br>Trial / Study Statistician:<br>Prof Amanda Lee<br>Professor of Medical Statistics,<br>Division of Applied Health Sciences,<br>Polwarth Building, Foresterhill, Aberdeen.<br>AB25 2ZD<br>Telephone 01224 437111<br>E-mail a.j.lee@abdn.ac.uk
Trial/Study Coordinating Centre:	Centre for Healthcare Randomised Trials (CHaRT)<br>3rd Floor, HSRU, Health Sciences Building,<br>Foresterhill<br>Aberdeen<br>AB25 2ZD<br><br>Telephone 01224 438140

CONTENTS

TRIAL / STUDY PERSONNEL AND CONTACT DETAILS 2
CONTENTS 5
PROTOCOL APPROVAL 7
LIST OF ABBREVIATIONS 8
SYNOPSIS 9
SUMMARY 12
1. INTRODUCTION 14
2. STUDY OBJECTIVES 18
2.1 OBJECTIVES 18
2.2 OUTCOMES 18
2.2.1 Primary Outcomes 18
2.2.2 Secondary Outcomes 18
3. STUDY DESIGN 19
4. STUDY POPULATION 20
4.1 NUMBER OF PARTICIPANTS 20
4.2 INCLUSION CRITERIA 21
4.3 EXCLUSION CRITERIA 22
4.4 NOTES ON INCLUSION AND EXCLUSION CRITERIA 22
5. PARTICIPANT SELECTION AND ENROLMENT 22
5.1 IDENTIFYING PARTICIPANTS 22
5.1.1 Primary care 22
5.1.2 Secondary care 23
5.2 CONSENTING PARTICIPANTS 24
5.3 SCREENING FOR ELIGIBILITY 24
5.4 INELIGIBLE AND NON-RECRUITED PARTICIPANTS 24
5.5 RANDOMISATION 24
5.5.1 Randomisation 24
5.5.2 Treatment Allocation 25
5.5.3 Emergency Unblinding Procedures 26
5.5.4 Withdrawal procedures 26
6. INVESTIGATIONAL MEDICINAL PRODUCT AND PLACEBO 27
6.1 STUDY DRUG 27
6.1.1 Study Drug Identification 27
6.1.2 Study Drug Manufacturer 27
6.1.3 Marketing Authorisation Holder 27
6.1.4 Labelling and Packaging 27
6.1.5 Storage 27
6.1.6 Summary of Product Characteristics 27
6.1.7 Accountability procedures 28
6.2 PLACEBO 28
6.3 DOSING REGIME 28
6.4 DOSE CHANGES 28
6.5 PARTICIPANT COMPLIANCE 30
6.6 OVERDOSE 30
6.7 OTHER MEDICATIONS 30
6.7.1 Permitted Medications 30
6.7.2 Prohibited Medications 30
7. STUDY ASSESSMENTS 31
7.1 SAFETY ASSESSMENTS 31
7.2 STUDY ASSESSMENTS 31
8. DATA COLLECTION 31
8.1 SCHEDULE FOR DATA COLLECTION 31
8.2 SOURCE DATA 34
9. STATISTICS, DATA ANALYSIS AND ECONOMIC EVALUATION 35
9.1 SAMPLE SIZE CALCULATION 35
9.2 PROPOSED ANALYSES 35
9.3 ECONOMIC EVALUATION 37
9.3.2 Collection of data 37

9.3.3 Participant Costs 37
9.3.4 NHS Costs of Health Services Used 37
9.3.5 Cost Effectiveness 38
10. ADVERSE EVENTS 38
10.1 DEFINITIONS 38
10.2 DETECTING ARs AND SAEs 39
10.3 RECORDING SAEs 39
10.4 EVALUATION OF SAEs 39
10.4.1 Assessment of Seriousness 39
10.4.2 Assessment of Causality 39
10.4.3 Assessment of Severity 40
10.4.4 Assessment of Expectedness 40
10.5 REPORTING OF SAEs / SARs / SUSARs 40
10.6 REGULATORY REPORTING REQUIREMENTS 41
10.7 FOLLOW UP PROCEDURES 41
11. PREGNANCY 41
12. TRIAL MANAGEMENT AND OVERSIGHT ARRANGEMENTS 41
12.1 PROJECT MANAGEMENT GROUP 41
12.2 TRIAL MANAGEMENT 41
12.3 TRIAL STEERING COMMITTEE 42
12.4 DATA MONITORING COMMITTEE 42
12.5 INSPECTION OF RECORDS 42
12.6 STUDY MONITORING 42
13. GOOD CLINICAL PRACTICE 42
13.1 ETHICAL CONDUCT OF THE STUDY 42
13.2 REGULATORY COMPLIANCE OF THE STUDY 42
13.3 INVESTIGATOR RESPONSIBILITIES 42
13.3.1 Informed Consent 43
13.3.2 Study Site Staff 43
13.3.3 Data Recording 43
13.3.4 Investigator Documentation 43
13.3.5 GCP Training 44
13.3.6 Confidentiality 44
13.3.7 Data Protection 44
14. STUDY CONDUCT RESPONSIBILITIES 44
14.1 PROTOCOL AMENDMENTS 44
14.2 PROTOCOL VIOLATIONS AND DEVIATIONS 44
14.3 STUDY RECORD RETENTION 45
14.4 END OF STUDY 45
14.5 CONTINUATION OF DRUG FOLLOWING THE END OF STUDY 45
15. REPORTING, PUBLICATIONS AND NOTIFICATION OF RESULTS 45
15.1 AUTHORSHIP POLICY 45
15.2 PUBLICATION 45
15.3 PEER REVIEW 45
REFERENCES 46
APPENDIX 1: RATIONALE FOR THE LOW DOSE THEOPHYLLINE STRATEGY 49
APPENDIX 2 : SUMMARY OF PRODUCT CHARACTERISTICS FOR THEOPHYLLINE 52
APPENDIX 3: PUBLICATION POLICY 57

PROTOCOL APPROVAL

A randomised, double-blind placebo controlled trial of the effectiveness of low dose oral theophylline as an adjunct to inhaled corticosteroids in preventing exacerbations of chronic obstructive pulmonary disease

TWICS (Theophylline With Inhaled Corticosteroid)

Signatures:

By signing this document I am confirming that I have read, understood and approve the protocol for the above study.

**Graham Devereux**<br>*Chief Investigator*	______________________<br>*Signature*	______________<br>*Date*
**Amanda Lee**<br>*Medical Statistics Team*	______________________<br>*Signature*	______________<br>*Date*
**Patricia Cooper**<br>*Clinical Trials Pharmacy*	______________________<br>*Signature*	______________<br>*Date*

LIST OF ABBREVIATIONS

AE	Adverse Event
AR	Adverse Reaction
bd	Twice daily
CHaRT	Centre for Healthcare Randomised Trials.
CI	Chief Investigator
CNS	Central Nervous System
COPD	Chronic Obstructive Pulmonary Disease
CRF	Case Reporting Form
CTA	Clinical Trial Authorisation
DSUR	Development Safety Update Report
EudraCT	European Union Drug Regulating Authorities Clinical Trials
FEV1	Forced expiratory volume in 1 second
FVC	Forced vital capacity
GCP	Good Clinical Practice
HDAC2	histone deacetylase 2
IB	Investigator's Brochure
IBW	Ideal body weight
ICS	Inhaled corticosteroid
IDMC	Independent Data Monitoring Committee
IMP	Investigational Medicinal Product
ISF	Investigator Site File
ISRCTN	International Standard Randomised Controlled Trial Number
LABA	Long acting β2 agonist
MHRA	Medicines and Healthcare products Regulatory Agency
NHS	National Health Service
NICE	National Institute for Clinical Excellence
OCS	Oral corticosteroid
od	Once daily
PDE	Phospho-diesterase
PIL	Patient Information Leaflet
QALY	Quality adjusted life year
R&D	Research and Development
REC	Research Ethics Committee
SAE	Serious Adverse Event
SAR	Serious Adverse Reaction
SPC	Summary of Product Characteristics
SUSAR	Suspected Unexpected Serious Adverse Reaction
TMF	Trial Master File
TSC	Trial Steering Committee
UAR	Unexpected Adverse Reaction

SYNOPSIS

Title	A randomised, double-blind placebo controlled trial of the effectiveness of low dose oral theophylline as an adjunct to inhaled corticosteroids in preventing exacerbations of chronic obstructive pulmonary disease.
Short title & Acronym	Theophylline With Inhaled CorticoSteroid (TWICS)
Co-Chief Investigator	Prof David Price<br>Prof Graham Devereux
Rationale	COPD airway inflammation is relatively insensitive to the anti-inflammatory effects of inhaled corticosteroids. Preclinical and pilot clinical trials indicate that theophylline at low dose (plasma concentration 1-5mg/l) increases the sensitivity of COPD airway inflammation to the anti-inflammatory effects of inhaled corticosteroids.
Objectives	To determine whether the addition of low dose oral theophylline (Uniphyllin MR 200mg od or bd [depending on ideal body weight and smoking status]) to inhaled corticosteroid (ICS) therapy in patients with COPD at high risk of exacerbation because of a history of at least two COPD exacerbations in the previous year:<br>• Reduces the risk of exacerbation.<br>• Is cost effective to the NHS.<br>• Improves quality of life.<br>• Improves lung function.<br>• Reduces health care use.<br>• Reduces mortality.
Trial Configuration	A pragmatic randomised, double-blind, placebo-controlled, multicentre clinical trial.
Setting	Both Primary & Secondary Care
Sample size estimate	With 669 subjects in each arm of the trial, the trial will be able to detect a 15% reduction in the number of exacerbations in the year of treatment (i.e. from an average of 2.22 to 1.89) with 90% power at the two-sided 5% significance level. Allowing for an estimated 6% loss to follow-up 712 subjects will be recruited in each trial arm: 1,424 in total
Number of participants	1,424 participants. More than 50% to be recruited from primary care.
Eligibility criteria	Inclusion criteria:<br>• Aged ≥ 40 years.<br>• A smoking history of at least 10 pack years ([average number of cigarettes/day x years smoked]/20).<br>• An established predominant respiratory diagnosis of COPD (GOLD/NICE Guideline definition: post bronchodilator FEV1/FVC<0.7).<br>• Current use of ICS therapy (irrespective of LABA and/or LAMA use).<br>• A history of at least two exacerbations requiring treatment with antibiotics and/or oral corticosteroid use in the previous year, based on patient report.<br>• Clinically stable with no COPD exacerbation for at least 4 weeks.

	• Able to swallow study medication.<br>• Able and willing to give informed consent to participate.<br>• Able and willing to participate in the study procedures; undergo spirometric assessment (see notes in section 4.2 about cases where spirometry may be contraindicated), complete study questionnaire.<br><br>Exclusion Criteria:<br>• Severe or unstable ischaemic heart disease.<br>• A predominant respiratory disease other than COPD.<br>• Any other significant disease/disorder which, in the investigator's opinion, either puts the patient at risk because of study participation or may influence the results of the study or the patient's ability to participate in the study.<br>• Previous allocation of a randomisation code in the study or current participation in another interventional study (CTIMP or non-CTIMP).<br>• Theophylline use currently.<br>• Known or suspected hypersensitivity to theophylline.<br>• Current use of drugs known to interact with theophylline and/or increase serum theophylline:<br>antimicrobials: aciclovir, clarithromycin, ciprofloxacin, erythromycin, fluconazole, ketoconazole, levofloxacin, norfloxacin;<br>cardiovascular: diltiazem, mexiletine, pentoxifylline, verapamil;<br>neurological: bupropion, disulfiram, fluvoxamine, lithium;<br>hormonal: medroxyprogesterone, oestrogens;<br>immunological: methotrexate, peginterferon alpha, tacrolimus;<br>miscellaneous: cimetidine, deferasirox, febuxostat, roflumilast, thiabendazole.[29]<br>• For women, current pregnancy or breast-feeding, or planned pregnancy during the study.
Description of interventions	Participants will be randomised to either low dose theophylline or an identical placebo. Dosage will be determined by the participants' ideal body weight and self-reported smoking status.<br><br>Non smoking participants:<br>theophylline 200mg once daily or placebo once daily<br><br>Participants who smoke with ideal body weight ≤60kg:<br>theophylline 200mg once daily or placebo once daily<br><br>Participants who smoke with ideal body weight >60kg:<br>theophylline 200mg twice daily or placebo twice daily<br><br>Further information about dosing is given in sections 6.3 and 6.4.
Duration of study	This study will last for 48 months. Each participant will be involved in the study for 12 months.

Randomisation and blinding	Determined by a computerised web-based randomisation system created by the University of Aberdeen's Centre for Healthcare Randomised Trials (CHaRT). Participants will be allocated with equal probability to intervention or control arms, stratified by centre, and recruitment setting (primary vs secondary care).
Outcome measures	Primary outcomes<br>• Total number of exacerbations of COPD necessitating changes in management (use of oral corticosteroids and/or antibiotics) during the one year treatment period.<br>• Cost-per-QALY gained during the one year treatment period.<br><br>Secondary outcomes:<br>• Total number of COPD exacerbations requiring hospital admission.<br>• Total number of episodes of pneumonia<br>• Total number of emergency hospital admissions (all causes).<br>• Disease specific health status (CATest; HARQ at some sites).<br>• Generic health related quality of life (EQ-5D).<br>• Lung function, post bronchodilator FEV1, FVC.<br>• Total inhaled corticosteroid dose/usage.<br>• Health care utilisation.<br>• Incremental cost-per-exacerbation avoided.<br>• Other adverse events.<br>• All cause and respiratory mortality.<br>• Modelled lifetime incremental cost per QALY.
Statistical methods	Baseline characteristics will be described for both treatment groups. The primary outcome of number of COPD exacerbations will be compared between randomised groups using negative binomial regression with length of time in the study as an offset. Estimates will then be adjusted for centre and other baseline covariates that are known to be strongly related to the outcome.

SUMMARY

Rationale: Chronic Obstructive Pulmonary Disease (COPD) is a progressive lung disease characterised by progressive airflow limitation. It affects approximately 3 million people in the UK, is the fifth leading cause of death in the UK and costs the NHS approximately £1 billion annually. Exacerbations of COPD account for 60% of NHS COPD costs and are associated with accelerated rate of lung function decline, reduced physical activity, reduced quality of life, increased mortality and increased risk of comorbidities such as acute myocardial infarction and stroke.

The majority of COPD treatment guidelines recommend inhaled corticosteroids (ICS), usually in combination with inhaled LABA to reduce exacerbation rates and improve lung function. However, compared to the marked response observed in asthma, COPD airway inflammation is relatively insensitive to the anti-inflammatory effects of ICS and even high doses fail to prevent exacerbations. In COPD, airway inflammation is driven by expression of multiple inflammatory genes, regulated by acetylation of core histones which open up the chromatin structure. Corticosteroids appear to work by reversing histone acetylation through the recruitment of histone deacetylase-2 (HDAC2) thereby switching off activated inflammatory genes. In COPD there is increased histone acetylation consequent upon markedly reduced HDAC2 activity/expression in airways, lung tissue and alveolar macrophages. It has been observed that the reduced HDAC2 activity of COPD can be reversed in a dose-dependent manner by theophylline at ‘low’ concentrations of 1-5mg/l. This basic research is supported by two small RCTs and a population based health administration database study.

Objectives: To determine the clinical effectiveness and cost-effectiveness of adding low dose theophylline (Uniphyllin MR 200mg od or bd [depending on smoking status and ideal body weight]) to ICS therapy in patients with COPD and a history of exacerbations treated with antibiotic and/or ICS.

The primary clinical outcome is the number of participant reported COPD exacerbations necessitating a change in management (minimum change treatment with antibiotics and/or oral corticosteroids) during the one year treatment period.
The primary economic outcome is the cost-per-QALY gained during the one year treatment period.

Method: A pragmatic randomised, double-blind, placebo-controlled, multicentre clinical trial.

Setting: Primary and Secondary care; General Practice and NHS hospitals.

Target population: 1424 patients with diagnosed COPD (post bronchodilator FEV1/FVC<0.7), currently receiving ICS as part of COPD treatment and with a history of ≥2 COPD exacerbations in the previous year requiring treatment with oral corticosteroids and/or antibiotics.

Main exclusion criteria: patients currently using theophylline or medications that increase theophylline serum levels.

Randomisation: When clinically stable subjects will be assessed, recruited and randomised with equal probability to intervention or control group.

Intervention: Intervention and control groups will receive usual NHS care: intervention group will also receive oral Uniphyllin MR at a dose predicted by pharmacokinetic modelling to achieve steady state serum concentration of 1-5mg/l for one year. Participants who do not smoke will receive 200mg od, participants who smoke will receive 200mg od if ideal body weight≤60kg and 200mg bd if ideal body weight>60kg. Control group will receive an identical oral placebo tablet od/bd (based on same weight and smoking criteria) for one year. Further information about dosing is provided in sections 6.3 and 6.4 of the protocol.

Assessment: Face to face health outcome assessments will be carried out for all participants at recruitment/baseline, 6, and 12 months, telephone contact will be made at approximately two weeks to ensure no adverse events and tolerating study medication. Participants will also be asked to record exacerbations and associated treatment and health service contact – space will be provided for this on the carton that study medication is shipped in. At 6 and 12 month follow-up, the following will be collected: history of exacerbations, health care utilisation, disease-related quality of life status (COPD assessment test [CATest]), MRC dyspnoea score, health related quality of life (EQ-5D), lung function (FEV1, FVC), adverse reactions and serious adverse events. The Hull Airways Reflux Questionnaire (HARQ) will be used at some sites to collect data on respiratory and GI symptoms.

Clinical relevance: Low dose theophylline is cheap (10p/day) and, if shown to make current ICS therapy more effective in a cost effective manner, it will improve the quality of life of COPD patients and reduce the burden of COPD on the NHS.

1. INTRODUCTION

COPD

COPD is a common lung disease[1,2] thought to affect around 3 million people in the UK, although only ~900,000 have been formally diagnosed.[2] The progressive airflow obstruction of COPD is associated with increasing disability, work absence, long-term morbidity, common physical and psychological co-morbidities, and premature mortality. COPD is the fifth leading cause of death in the UK, accounting for about 5% of all deaths. COPD costs the NHS about £1 billion per year, 60% of which is accounted for by exacerbations.[3]

Exacerbations are an important clinical feature of COPD, they are associated with accelerated rate of lung function decline,[4] reduced physical activity,[5] reduced quality of life[6] increased mortality[7] and increased risk of comorbidities such as acute myocardial infarction and stroke. Hospital admissions precipitated by COPD exacerbations have steadily increased and are the second leading cause for emergency hospital admission[1]. COPD is one of the most costly 'inpatient' conditions treated by the NHS.[1,2] Over 30% of patients admitted to hospital with COPD are readmitted within 30 days and an average of 12% of COPD patients die in the year following admission to hospital with an exacerbation.[7]

Rationale for investigating low dose theophylline

Most international COPD management guidelines [1,8] recommend the use of ICS, usually in combination with inhaled LABA, to reduce COPD exacerbation rates and improve lung function and quality of life. However, when compared to the marked responses observed in asthma, ICS in COPD fail to fully suppress airway inflammation and patients continue to have exacerbations despite high ICS doses. Furthermore little or no positive impact of ICS on mortality or disease progression is evident.[9,10] Although ICS are beneficial in COPD a relative insensitivity of COPD airway inflammation to the anti-inflammatory effects of high dose ICS has been demonstrated.[11-13] The molecular mechanisms contributing to this reduced corticosteroid sensitivity in COPD are the basis for this trial. The chronic airway inflammation of COPD is driven by expression of pro-inflammatory genes regulated by acetylation of core histones which open up the chromatin structure, enabling gene transcription.[14] Histone acetylation is reversed by histone deacetylase (HDAC) enzymes. However in COPD, airway HDAC2 activity/expression is markedly reduced and there is increased acetylation of core histones associated with pro-inflammatory genes.[15] Corticosteroids appear to work by reversing histone acetylation through the recruitment of HDAC2, [14,16,17] thereby switching off activated pro-inflammatory genes. The reduced HDAC2 activity observed in COPD is thought to contribute to the reduced sensitivity of COPD airway inflammation to corticosteroids.[15-18]

It has been observed that the reduced HDAC2 activity of COPD can be reversed by 'low dose' theophylline (blood levels of 1-5mg/l)[19,20], reducing corticosteroid insensitivity in COPD. A synergism exists between 'low dose' theophylline and corticosteroids in suppressing the release of inflammatory mediators in COPD airways[19,20]. These in vitro findings have been supported by the results of two small RCTs[21,22] and a health administration database study[23] suggesting that theophylline exerts a protective effect against COPD exacerbations. The first RCT in 35 patients with acute COPD exacerbations found that low- dose theophylline increased responsiveness to corticosteroids as measured by increased HDAC activity and further reduced concentrations of pro-inflammatory mediators in induced sputum compared to inhaled corticosteroids alone.[21] In the second (n=30) pilot RCT of COPD patients, the combination of low dose theophylline with high dose ICS was associated with increased HDAC, improved lung function and reduced sputum inflammatory cells and mediators,

whereas either drug alone was ineffective.[22] A Canadian health administration database study of 36,492 COPD patients reported that treatment with theophylline either alone or in combination with ICS was more protective against exacerbations than treatment with LABA or ICS-LABA.[23]

Oral theophylline has conventionally been used as a bronchodilator in COPD, however in order to achieve modest clinical effects relatively high blood levels (10-20mg/l) are required. The bronchodilator effect of 'high dose' theophylline is the consequence of inhibition of phosphodiesterase (PDE) and consequent relaxation of airway smooth muscle, however PDE inhibition is also associated with the well recognised side effects of theophylline, namely nausea, gastro-intestinal upset, cardiac arrhythmias and malaise. The narrow therapeutic index, modest clinical effect, side effect profile, drug interactions, the need for blood level monitoring and the availability of more effective inhaled therapies has resulted in current COPD guidelines relegating 'high dose' theophylline to third line therapy.[1]

This trial builds on the emerging evidence from molecular, animal, and clinical studies that 'low dose' (1-5mg/l) theophylline may produce a beneficial synergistic effect in COPD by increasing the corticosteroid sensitivity of the airway inflammation underlying COPD. It is anticipated that 'low dose' theophylline will restore corticosteroid responsiveness in COPD patients, and when used in combination with ICS will reduce the rate of COPD exacerbation.

Hypothesis

The hypothesis being tested is that the addition of 'low dose' theophylline to ICS therapy in COPD reduces the risk of COPD exacerbation requiring treatment with antibiotics and/or OCS during the year of treatment, delivers quality of life improvements and is cost-effective.

Dosage

The in vitro studies outlined above demonstrate the critical importance of serum theophylline concentration, with serum levels 1-5 mg/l having the maximal effect on reducing corticosteroid insensitivity whereas at levels >10mg/l theophylline is inhibitory, augmenting corticosteroid insensitivity. Previous studies of low dose theophylline have used a "one size fits all" approach to dosing e.g. all participants receive 100mg bd or 200mg bd, and inevitably some participants will have had >10mg/l theophylline levels associated with an increased likelihood of side effects and lack of therapeutic effect.

In this trial theophylline dosing is based upon pharmacokinetic modeling [24,25] of theophylline incorporating the major determinants of theophylline steady state concentration, i.e. weight, smoking status, clearance of theophylline (low, normal, high), and is designed to achieve a steady state (Css) serum theophylline of 1-5 mg/l and to certainly be <10mg/l, (>10mg is the level associated with high dose theophylline, possible side effects and augmentation of corticosteroid insensitivity). Full details are appended in Appendix 1.

The dosing of both the experimental (Uniphyllin MR 200mg tablets) and control placebo interventions will be determined by the participants ideal body weight and self-reported smoking status and is outlined below. Ideal body weight will be used unless the actual weight is lower than the ideal body weight. Intervention will be for one year.

Smokers ideal body weight ≤60kg & non-smokers
Theophylline 200mg od or placebo od

Smokers ideal body weight >60kg
Theophylline 200mg bd or placebo bd

At the outset of the study, to be classed as a "non-smoker" a participant must have abstained from smoking for 12 weeks or more. A participant who has given up smoking recently (less than 12 weeks ago) will – at the outset of the study - be classed as a smoker. If the actual body weight is lower than the ideal body weight, actual body weight will be used to determine dose.

Ideal body weight (IBW) will be calculated by

- IBWfemale = 45 + 0.9(height in cms – 152) Kg
- IBWmale = 50 + 0.9(height in cms – 152) Kg

Changes in smoking status:
Non-smoking participants who start smoking will reduce serum theophylline over 3-4 months because of the timecourse of liver enzyme induction. An increase in dosing to 200mg bd will be required for subjects IBW >60kg.

Smoking participants who stop smoking will increase serum theophylline over 3-4 months because of the timecourse of induced liver enzymes to return to normal. For smokers IBW 40-60kg smoking cessation will not increase serum levels to >10mg/l. No change in dose will be required.

For smokers weighing IBW>60kg smoking cessation will require a dose reduction to 200mg od. If dose is not reduced, for subjects IBW 60-70kg a small minority (3-5%) who are slow clearers of theophylline, smoking cessation will increase serum theophylline to 11mg/l that may be associated with side effects. We anticipate this may be an issue for 1-2 subjects.

More detailed information about patients who change their smoking status during their participation in the trial is given in section 6.4 of the protocol.

Potential risks
Theophylline has been used for more than 70 years to treat asthma and COPD and has a well documented safety profile. Adverse reactions may occur with serum theophylline >10mg/l, the usual therapeutic range for 'high dose' theophylline being 10-20mg/l. These reactions are discussed in more detail in section 10.4.4, but can be broadly grouped into immunological (analphylactic, anaphylactoid reactions), metabolic (hyperuricaemia) psychiatric (agitation, anxiety, insomnia), CNS (convulsions, dizziness, headache, tremor), cardiac (atrial/sinus tachycardia, palpitations), gastrointestinal (abdominal pain, diarrhoea , gastric irritation, GOR, nausea, vomiting) , dermatological (pruritus, rash) and renal/urinary (diuresis, urinary retention). For more detailed information on the potential risks, special warning and precautions for use of theophylline please refer to the Summary of Product Characteristics (Appendix 2).

A Cochrane Review of 'high dose' theophylline therapy for COPD concluded that 8% of participants experience side effects potentially attributable to theophylline.[26] Two studies of 'low dose' theophylline each with nearly 4000 subjects reported that dosing with theophylline 200mg-400mg/day is associated with a side effect rate of 4.7-4.8%[27,28]. These

data suggest that in this trial ~35 subjects will experience side effects, however it is anticipated that tailoring of dosing to weight and smoking status will ensure serum theophylline levels <10mg/l and will be associated with an appreciably lower rate of theophylline associated side effects.

Theophylline in the form of intravenous aminophylline has traditionally been used in the treatment of severe acute exacerbations of COPD in the hospital setting. Research does not support the use of intravenous aminophylline[24] and NICE COPD Guidelines include a Grade D recommendation on the use of intravenous aminophylline in acute severe exacerbations and also recommends the measurement of serum theophylline 24 hours after commencing intravenous aminophylline. The clinical protocol for use of intravenous aminophylline was established during the era of 'high dose' oral theophylline when patients would be admitted with serum levels 10-20mg/l and a loading dose of aminophylline would raise serum theophylline levels to toxic levels (>20mg/l). For a patient not established on oral theophylline the intravenous protocol comprises a bolus of intravenous aminophylline (usually 250mg, or 5mg/kg) followed by a maintenance dose (0.5mg/kg/hr), whereas for a patient established on oral theophylline the bolus dose is omitted (because of concerns regarding toxicity) and a maintenance infusion (0.5mg/kg/hr) commenced. In the era of 'high dose' theophylline it was critical to establish if a patient was taking oral theophylline before a physician commenced a patient on intravenous aminophylline.

Inevitably during this trial some participants will be hospitalised with life threatening exacerbations of COPD (a secondary outcome) and very occasionally the caring physician may wish to use intravenous aminophylline. Although rare, this acute scenario does require consideration. Pharmacokinetic modelling demonstrates that a 250mg (or 5mg/kg if <50kg) loading dose of aminophylline can be administered to trial participants and their serum theophylline will remain within the therapeutic 'high dose' bronchodilating level of 10-20mg/l (Appendix 1). As per NICE recommendations serum theophylline will need to be measured 24 hours after commencing intravenous aminophylline (allocation status will not be discernable from such a level).

Participants inadvertently taking a second tablet in a day because they forgot they had taken one already will not come to harm because of the low steady state serum concentration. Deliberate overdosing can be associated with serious consequences (see section 6.6).

Known and potential benefits

Theophylline has Marketing Authorisation and has been shown to be efficacious for the treatment and prophylaxis of bronchospasm associated with asthma and COPD. It is anticipated participants of this trial allocated to 'low dose' theophylline with serum levels 1-5mg/l will benefit from a reduced rate of COPD exacerbations while avoiding the side effects associated with traditional high bronchodilating theophylline doses (serum levels 10-20mg/l). Participants allocated to placebo will have an unchanged rate of exacerbation.

Exacerbations of COPD are associated with an adverse prognosis, increased mortality, and a reduced quality of life, moreover COPD exacerbations are the most costly aspect of COPD to the NHS, accounting for 60% of total NHS COPD direct costs. A reduction in COPD exacerbations by cheap (10p/day) 'low dose' theophylline will benefit patients, society and the NHS.

2. STUDY OBJECTIVES

2.1 OBJECTIVES

The primary objective of this trial is to determine the clinical and cost-effectiveness of adding 'low dose' theophylline (Uniphyllin MR 200mg od or bd [depending on ideal body weight and smoking status]) to inhaled corticosteroid (ICS) therapy in patients with COPD and a history of two or more exacerbation treated with antibiotic and/or oral corticosteroids in the previous year.

The secondary objectives are:

- To compare hospital admissions with a primary diagnosis of exacerbation of COPD.
- To compare total number of episodes of pneumonia.
- To compare total number of emergency hospital admissions.
- To compare lung function.
- To compare all-cause and respiratory mortality.
- To compare drug reactions and serious adverse events.
- To compare health related quality of life.
- To compare disease specific health status.
- To compare total inhaled corticosteroid dose/usage
- To compare health care utilisation.
- To compare incremental cost-per-exacerbation avoided
- To assess lifetime cost-effectiveness based on extrapolation modelling.
- To compare modelled lifetime incremental cost per Quality Adjusted Life Year

2.2 OUTCOMES

2.2.1 Primary Outcomes

The primary outcome measure will be the total number of exacerbations of COPD necessitating changes in management (minimum management change - use of oral corticosteroids and/or antibiotics) during the one year treatment period, as reported by the participant.

The primary economic outcome measure will be cost-per-QALY gained during the one year treatment period.

2.2.2 Secondary Outcomes

- Total number of COPD exacerbations requiring hospital admission.
- Total number of episodes of pneumonia.
- Total number of emergency hospital admissions (all causes).
- Lung function (FEV1, FVC) post bronchodilator using spirometry performed to ATS/ERS standards.
- All-cause and respiratory mortality.
- Serious adverse events, adverse reactions.
- Total dose of inhaled corticosteroid.
- Utilisation of primary or secondary health care for respiratory events
- Disease specific health status using the COPD Assessment Test (CAT); MRC dyspnoea scale
- Change in disease associated symptoms using the Hull Airways Reflux Questionnaire (HARQ) [*this will only be done at some recruitment sites*].

- Generic health related quality of life using EuroQoL 5D (EQ-5D) Index.
- Modelled lifetime incremental cost per Quality Adjusted Life Year.

Further information on the timing and methods used to collect study endpoints is provided in section 7.1 Study Assessments.

3. STUDY DESIGN

The study is a pragmatic double blind randomised, placebo-controlled, UK multicentre clinical trial comparing the addition of low dose theophylline or placebo for one year to current COPD therapy that includes ICS, in patients with COPD who have had two or more exacerbations of COPD in the previous year treated with oral corticosteroids and/or antibiotics. Figure 1 provides a schematic representation of study design.

Figure 1: Study design

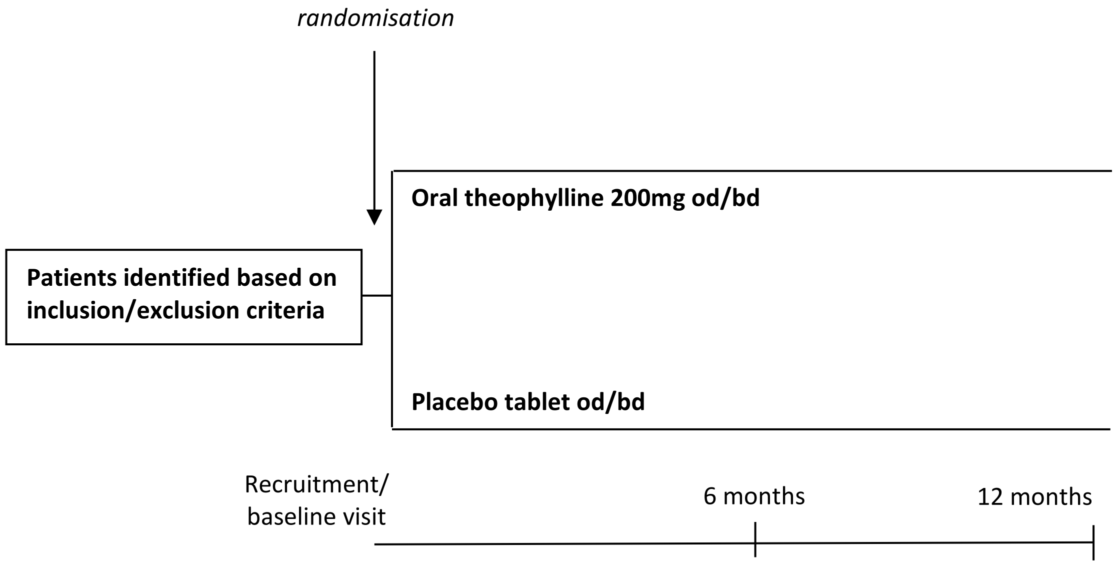

Face-to-face study assessments will be carried out in all subjects at recruitment/baseline, 6, and 12 months as shown in Figure 2.

Figure 2: Flow diagram of study schedule

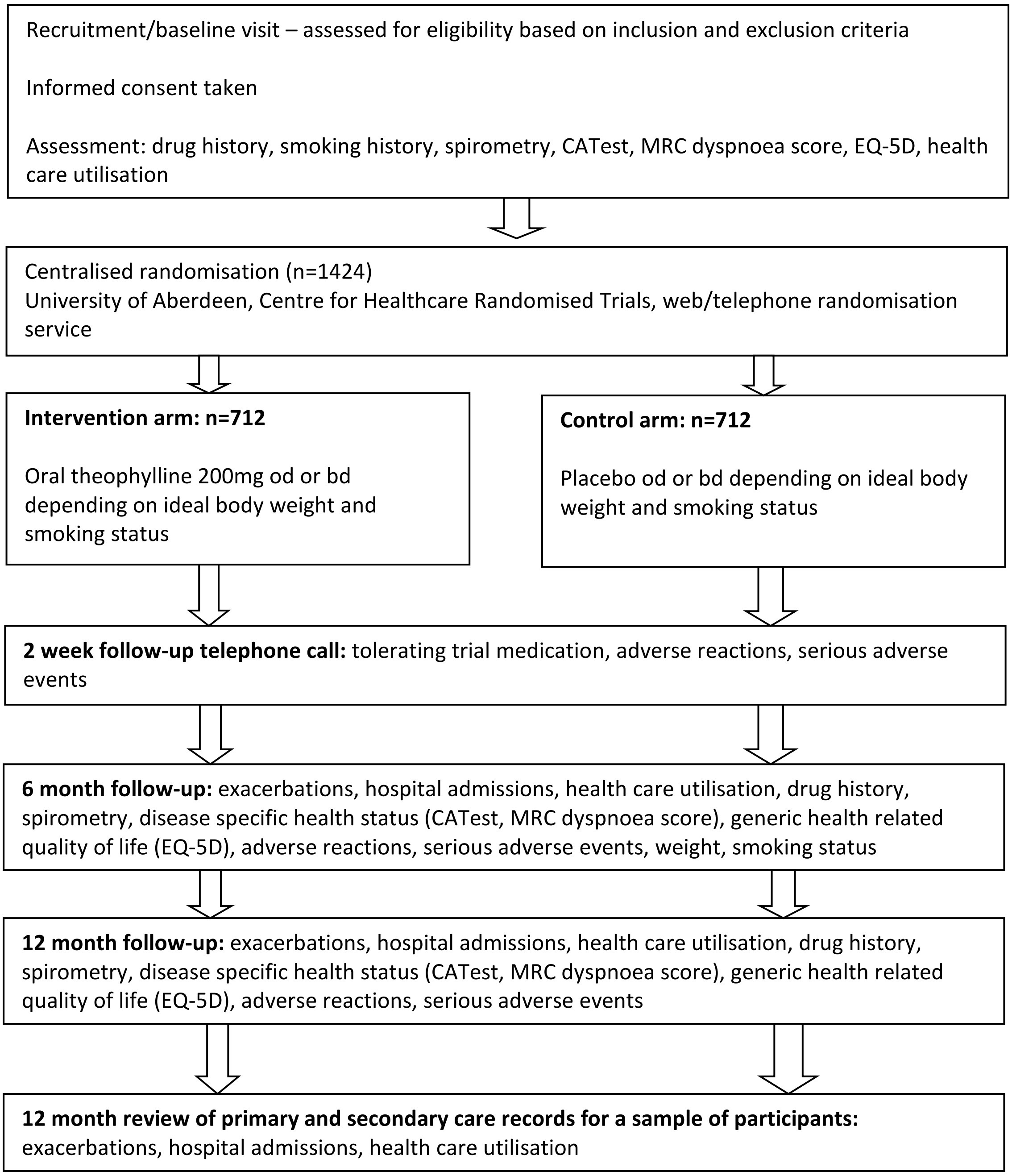

4. STUDY POPULATION

4.1 NUMBER OF PARTICIPANTS

The start of funding for the trial is 1 July 2013.

Recruitment will be for two years, each subject will spend one year in the trial.

The trial will recruit a total of 1,424 participants; 712 in each treatment arm.

We anticipate seven participating sites (Aberdeen, Birmingham, Glasgow, Hull, Liverpool,

Newcastle and Norwich). Additional sites may be added if recruitment falls short in these sites.

It is anticipated that each participating site will recruit an average of 203 patients, although there is scope for over-recruitment at some sites to compensate for potential under-recruitment at other sites.

4.2 INCLUSION CRITERIA

Eligibility for inclusion in the trial will be confirmed by a medically qualified person and recorded in the medical notes.

Patients will be enrolled if they meet all of the following criteria:

- Aged ≥ 40 years.
- A smoking history of at least 10 pack years ([average number of cigarettes/day x years smoked]/20).
- An established predominant respiratory diagnosis of COPD (GOLD/NICE Guideline definition: post bronchodilator FEV1/FVC<0.7).
- Current use of ICS therapy (irrespective of LABA and/or LAMA use).
- A history of at least two exacerbations requiring treatment with antibiotics and/or oral corticosteroid use in the previous year, based on patient report.
- Clinically stable with no COPD exacerbation for at least 4 weeks.
- Able to swallow study medication.
- Able and willing to give informed consent to participate.
- Able and willing to participate in the study procedures; undergo spirometric assessment*, complete study questionnaire.

* There are a number of surgical procedures and other conditions after which spirometry is not recommended for a period of time. The table below is a pragmatic conservative adaptation of the recommendations of Cooper (2010). Other clinical or personal circumstances may preclude spirometry in individual cases. Such cases should be discussed with the study team.

In cases where spirometry is contraindicated, it is acceptable to inclue the patient on the basis of a prior diagnosis of COPD that is based on spirometry.

**Surgical procedure/condition**	**Notes about spirometry**
Thoracic/abdominal surgery	3 months post surgery
Brain, eye, ear, ENT surgery	3 months post surgery
Pneumothorax	3 months post resolution
Myocardial infarction	3 months
Ascending aortic aneurysm	3 months post repair
Haemoptysis	1 month post free of haemoptysis
Pulmonary embolism	Safe if on anticoagulation
Angina	Safe if stable
Severe hypertension (systolic >200 mm Hg, diastolic >120 mm Hg):	Measure blood pressure if suspected

Cooper (2010). An update on contradindicatoions for lung fuction testion. *Thorax* doi:10.1136/thx.2010.139881

4.3 EXCLUSION CRITERIA

The following exclusion criteria will apply:

- Severe or unstable ischaemic heart disease.
- A predominant respiratory disease other than COPD.
- Any other significant disease/disorder which, in the investigator's opinion, either puts the patient at risk because of study participation or may influence the results of the study or the patient's ability to participate in the study.
- Previous allocation of a randomisation code in the study or current participation in another interventional study (CTIMP or non-CTIMP).
- Theophylline use currently.
- Known or suspected hypersensitivity to theophylline.
- Current use of drugs known to interact with theophylline and/or increase serum theophylline:

  <u>antimicrobials:</u> aciclovir, clarithromycin, ciprofloxacin, erythromycin, fluconazole, ketoconazole, levofloxacin, norfloxacin;
  <u>cardiovascular:</u> diltiazem, mexiletine, pentoxifylline, verapamil; neurological: bupropion, disulfiram, fluvoxamine, lithium;
  <u>hormonal:</u> medroxyprogesterone, oestrogens;
  <u>immunological:</u> methotrexate, peginterferon alpha, tacrolimus;
  <u>miscellaneous:</u> cimetidine, deferasirox, febuxostat, roflumilast, thiabendazole.[29]
- For women, current pregnancy or breast-feeding, or planned pregnancy during the study.

4.4 NOTES ON INCLUSION AND EXCLUSION CRITERIA

Short or long term use of azithromycin is not an exclusion criterion.

Patients with stable angina, stable heart failure, previous MI but now stable, are not excluded.

Topical oestrogens or aciclovir are not an exclusion criteria.

Nicotine replacement is not an exclusion criterion.

Patients with Alpha-1-Antitrypsin Deficiency and COPD should be excluded because the predominant diagnosis is Alpha-1-Antitrypsin Deficiency.

5. PARTICIPANT SELECTION AND ENROLMENT

5.1 IDENTIFYING PARTICIPANTS

Potential participants will be recruited from both primary and secondary care across the centres; however we envisage the majority of participants (>50%) will be recruited within primary care. Recruitment strategies will differ between centres depending on local geographic and NHS organisational factors.

5.1.1 Primary care

In England recruitment from General Practices will be conducted in conjunction with the Primary Care Research Network/Comprehensive Local Research Network. The Lead CLRN (Northumberland, Tyne & Wear CLRN) will be responsible for facilitating the set-up of the

study within primary care and will liaise with the relevant local PCRNs based within the collaborating centres region.

For General Practices acting as Participant Identification Centres (PICs), the local PCRN/collaborating centre will liaise directly with practice managers/GPs who will perform a database search (based on search criteria including one exacerbation treated with oral corticosteroids in previous year, interacting medications) to identify potential participants. Potentially suitable participants will be sent an invitation letter on practice headed paper and a patient information leaflet (PIL) informing them of the trial aims and level of participation required. The letter will provide a range of methods for interested potential participants to contact the local trial team (telephone, text, e-mail, reply paid envelope) for more information and to arrange a recruitment visit should the participant be agreeable. GP records will be screened on two occasions at least a year apart.

For General Practices acting as independent study sites, the local PCRN/collaborating centre will liaise directly with GP practice managers/GPs who will perform a database search (based on search criteria described above) to identify potential participants. Potentially suitable participants will be sent an invitation letter on practice headed paper and a PIL informing them of the trial aims and level of participation required. The letter will provide a range of methods for interested potential participants to contact the local general practice based trial team (telephone, text, e-mail, reply paid envelope) for more information and to arrange a recruitment visit should the participant be agreeable. GP records will be screened on up to two occasions at least a year apart.

In Scotland the Scottish Primary Care Research Network will mirror the role undertaken by the English PCRN by identifying potential participants in primary care.

In some centres COPD Community Matrons and other Intermediate Care services for patients with COPD are available. Potentially suitable participants will be sent an invitation letter on headed paper and a PIL informing them of the trial aims and level of participation required. The letter will provide a range of methods for interested potential participants to contact the local trial team (for example telephone, text, e-mail, reply paid envelope) for more information and to arrange a recruitment visit should the participant be agreeable.

Patients with COPD attending or who have attended Pulmonary Rehabilitation classes will be sent letter an invitation letter on headed paper and a PIL informing them of the trial aims and level of participation required. The letter will provide a range of methods for interested potential participants to contact the local trial team (for example telephone, text, e-mail, reply paid envelope) for more information and to arrange a recruitment visit should the participant be agreeable.

Recruitment in primary care will be supplemented by posters located in General Practice waiting areas and Community Pharmacies.

Other potential avenues for identifying eligible patients include smoking cessation clinics, community spirometry clinics and other services provided in primary or secondary care for patients with COPD. As above, potentially eligible patients will be provided with an invitation letter on headed paper and PIL.

5.1.2 Secondary care

Potential participants will also be identified from patients who are attending (or who have

previously attended) Respiratory Out-Patient appointments at the hospitals of the individual recruiting centres. The first contact will be made by a member of the care team within the clinic; who will provide a brief overview of the study, a letter of invitation on headed paper and a PIL informing them of the trial aims and level of participation required. The letter will provide a range of methods for interested potential participants to contact the local trial team (for example telephone, text, e-mail, reply paid envelope) for more information and to arrange a recruitment visit should the participant be agreeable.

If a trial centre has access to a Volunteer Database/Registry; participants (who meet the essential study eligibility criteria) will be identified and contacted via telephone/letter by a member of the research team. A letter of invitation on headed paper and PIL will be sent to the potential participant informing them of the trial aims and level of participation required. The letter will provide a range of methods for interested potential participants to contact the local trial team (for example telephone, text, e-mail, reply paid envelope) for more information and to arrange a recruitment visit should the participant be agreeable.

5.2 CONSENTING PARTICIPANTS
Informed consent to participate in the trial will be sought and obtained according to Good Clinical Practice (GCP) guidelines. Informed signed consent forms will be obtained from the participants by an appropriately trained individual. Potential participants will be given sufficient time to accept or decline involvement and will be given opportunity to ask questions. It will be explained that entry into the trial is entirely voluntary and that treatment and care will not be affected by their decision. It will also be explained that they can withdraw at any time. In the event of their withdrawal it will be explained that their data collected so far cannot be erased and we will seek consent to use the data in the final analyses where appropriate. Participants who cannot give informed consent (e.g. due to their mental state) will be not be eligible for participation.

5.3 SCREENING FOR ELIGIBILITY
Before entry into the trial, potential participants will be screened for the following:

- Confirmation of COPD diagnosis.
- Patient meets inclusion criteria detailed in section 4.2.
- Patient does not fulfil any exclusion criteria detailed in section 4.3.

5.4 INELIGIBLE AND NON-RECRUITED PARTICIPANTS
Brief details of all screened patients will be recorded (including – where known – age, sex and smoking status). If the patient was not eligible, the reason for this will be noted. If the patient was eligible, but declined to take part, this will be noted.

Patients not recruited to the study will remain on their existing treatment.

5.5 RANDOMISATION

5.5.1 Randomisation
A computerised randomisation system created by CHaRT will allocate participants with equal probability to intervention or control arms, stratified by centre, and recruitment setting (primary vs secondary care). This randomisation application will be available 24 hours a day, 7 days a week as both an Interactive Voice Response (IVRS) telephone system and as an internet based application.

To ensure double blinding, study medication/placebo will be identical in terms of

appearance, taste, touch and smell and dispensed in numbered boxes of identical appearance and labelling.

When informed of their patient's participation in the trial, GPs will be advised to manage their patient for exacerbations as per normal clinical practice and that they are to assume the participant is taking low dose theophylline and the prescription of interacting drugs should be avoided. In the event that drugs that interact to increase theophylline concentration have to be prescribed for 3 weeks or less, patients will be asked to suspend taking study medication and recommence their study medication after the course of interacting drug has been completed.

If the interacting drug is prescribed for more than 3 weeks, participants will discontinue the study medication but remain in the study and be followed up in accordance with the trial protocol. In these cases, participants will be asked to return unused study medication; no further supplies will be given.

Participants will be given (and advised to carry) a credit card sized alert card giving brief information about the trial and advice for clinicians, contact details for the local investigator, and the contact details for emergency unblinding.

At the time of recruitment (and within the PIL) participants will be advised to inform the study team if they stop or start smoking as this may necessitate a change in dosing as outlined in section 1. Failure of the participant to notify the trial team of smoking cessation will not result in serum theophylline rising to toxic levels. Smoking status will be checked at assessment visits.

5.5.2 Treatment Allocation
Participants will be randomised to theophylline or placebo. The first bottle (four week supply) of study medication (or placebo) will be provided to the participant via a participating Clinical Trials Pharmacy.

Each participant will receive two further supplies of six bottles (each bottle being a four week supply). These supplies will be delivered to participants via a courier service (or other signed for delivery service) operated by a third party. These shipments will be made around week 3 and week 27 to enable continuity of supply. Receipt of trial medication will be confirmed by signature on receipt.

Written informed consent to pass on participant's name and address to the third party distributer will be obtained.

The medication label will inform participants of dosing. Each supply pack will contain information on interacting drugs and side effects, and the need to inform the trial centre of relevant information in relation to these, or any changes to smoking habits.

After randomisation, we will inform the participant's GP of their participation in the study. If the participant has nominated a "best contact", we will also write to the best contact to confirm their willingness to act as such. We collect best contact information in case we cannot contact the participant themselves – for example if they have moved house or are in hospital.

5.5.3 Emergency Unblinding Procedures

The decision to unblind a single case should be made when knowledge of an individual's allocated treatment is required to:

- to enable treatment of severe adverse event/s, or
- in the event of an overdose
- to enable reporting of a SUSAR

Where possible, requests for emergency or unplanned unblinding of individuals should be made via the Trial Manager based in CHaRT at the University of Aberdeen. Agreement of either of the co-Chief Investigators will then be sought. However, in circumstances where there is insufficient time to make this request or for agreement to be sought, the treating clinician should make the decision to unblind immediately.

Unblinding will be done via the IVRS. In the event of failure of the IVRS (for example if the server is down), unblinding will be conducted by the on-call pharmacist based at Aberdeen Royal Infirmary who can be obtained by the switchboard (0845 456 6000).

All instances of unblinding should be recorded and reported in writing to CHaRT by the local investigator, including the identity of all recipients of the unblinding information.

Allocation should not routinely be revealed to CHaRT personnel, the co-Chief Investigators, or members of the research team at the site. In the event of unblinding to report a SUSAR, it is anticipated that one of the co-Chief Investigators (DP) will take responsibility for unblinding and reporting.

5.5.4 Withdrawal procedures

Patients may be withdrawn from treatment for any of the following reasons:

- Patient withdraws consent for treatment.
- Unacceptable adverse effects.
- Intercurrent illness preventing further treatment.
- Development of serious disease preventing further treatment or any change in the patient's condition that justifies the discontinuation of treatment in the clinician's opinion.
- Patient is prescribed a contra-indicated medicine for longer than 3 weeks (see section 6.7.2)

If participants are withdrawn from treatment they will be encouraged to remain in the trial and be followed up as per trial schedule or, failing this, to allow routine follow-up data to be used for trial purposes (hospital/GP medical records).

In cases where a participant withdraws from study treatment, but wishes to be prescribed low-dose theophylline, their GP can be advised of this by letter. If the GP considers this appropriate, they may prescribe theophylline.

Participants who wish to withdraw from study follow-up should be encouraged to allow routine follow-up data to be used for trial purposes (hospital/GP medical records).

Sites should encourage participants withdrawing from treatment to return all unused study medication to the local study centre, where destruction can be organised via Clinical Trials Pharmacy.

6. INVESTIGATIONAL MEDICINAL PRODUCT AND PLACEBO

6.1 STUDY DRUG

6.1.1 Study Drug Identification
Theophylline (Uniphyllin® Continus®) 200mg.

6.1.2 Study Drug Manufacturer
Uniphyllin MR 200mg tablets will be supplied by Napp Pharmaceuticals Limited, Cambridge Science Park, Cambridgeshire, CB4 0GW.

6.1.3 Marketing Authorisation Holder
The marketing authorisation holder is Napp Pharmaceuticals Limited, Cambridge Science Park, Cambridgeshire, CB4 0GW. Marketing Authorisation number: PL 16950/0066-0068

6.1.4 Labelling and Packaging
The manufacturer and its associated companies will arrange to label and package the study medication. The product will be provided in tamper proof bottles containing one month's supply of medication (28 or 56 tablets depending on dosing). Bottles will be colour coded for the once or twice daily dosing regimes. Single bottles will be delivered to clinical trial pharmacies in order that the first four week supply can be provided to participants from the local clinical trial pharmacy. [*Patients recruited from GP practices acting as independent study sites will receive their first four week supply via courier or other signed for delivery service, with signature on receipt.*] Remaining bottles will then be packed into supply boxes containing six bottles prior to shipment to participants.

6.1.5 Storage
The storage advice for theophylline is "Do not store above 25°C".

After manufacture, drugs will be shipped to, and stored by, the third party distributer. Drugs stored at the manufacturer and the distributer will be temperature monitored, with any drug packs subject to temperature deviations reported to the trial office and quarantined immediately. A decision will be taken as to whether affected drug packs are safe to use and can be removed from quarantine, or whether they should be destroyed.

Drug packs will be sent by courier (or other signed for delivery service) to participants, with signature on receipt. Participants will be advised to store their medication below 25°C but there will be no temperature monitoring after dispatch from the third party.

6.1.6 Summary of Product Characteristics
The Summary of Product Characteristics (SmPC) is given in Appendix 2.

Uniphyllin® continus® 200mg, 300mg and 400mg are licensed for the treatment and prophylaxis of bronchospasm associated with asthma, chronic obstructive pulmonary disease and chronic bronchitis. They are also indicated for the treatment of left ventricular and congestive cardiac failure. This current study will not be administering theophylline outside of its licensed indications.

The expected side effects of theophylline are presented in section 10.4.4.

6.1.7 Accountability procedures

Clinical trial supplies will only be delivered to trial sites once full ethical and regulatory approvals have been granted, and all local approvals are in place. This will be confirmed by the Trial Manager acting on behalf of the study sponsor. Recruitment will be monitored centrally to ensure sufficient medication is manufactured/available at clinical trials pharmacies and at the distributer. Shipment receipts will be provided to the trial office.

Participants will be asked to return unused study medication at their six and twelve month visits. Returned medication will be subject to tablet count and returned to the local Clinical Trials Pharmacy for destruction. If drug packs are not returned, participants will be advised to return to their local pharmacy for destruction or to dispose of safely.

6.2 PLACEBO

Placebo tablets will be manufactured to look identical to Uniphyllin MR 200mg tablets by Napp Pharmaceuticals Limited, Cambridge Science Park, Cambridgeshire, CB4 0GW.

6.3 DOSING REGIME

The dosing of both the active and control interventions will be determined by the participant's ideal body weight and self-reported smoking status as shown below.

Treatment duration is 12 months.

Table 4 Dosing regimen

Smokers ideal body weight ≤60kg & non-smokers<br>Theophylline 200mg od or placebo od<br><br>*Smokers ideal body weight >60kg*<br>Theophylline 200mg bd or placebo bd<br><br>At the outset of the study, to be classed as a "non-smoker" a participant must have abstained from smoking for 12 weeks or more. A participant who has given up smoking recently (less than 12 weeks ago) will – at the outset of the study – be classed as a smoker. If the actual body weight is lower than the ideal body weight, actual body weight will be used to determine dose.

6.4 DOSE CHANGES

In the event that drugs that interact to increase theophylline concentration have to be prescribed for three weeks or less the study medication will be suspended and recommenced after the course of interacting drug has been completed.

If the interacting drug is prescribed for more than 3 weeks, participants will discontinue the study medication (they will be asked to return any unused medication, no further supplies will be provided) but remain in the study and be followed up in accordance with the trial protocol.

Participants who are hospitalised with life threatening COPD exacerbations may be treated with intravenous aminophylline. Study medication will have to be suspended whilst the participant receives intravenous aminophylline and restarted when aminophylline is discontinued, and as per NICE recommendations serum theophylline will need to be measured 24 hours after commencing intravenous aminophylline (allocation status will not be discernable from such a level). This will be detailed on the participants credit card sized

alert card and the trial website.

Changes in dosing during the trial may be required. These are summarised in Table 5.

Table 5 – changes in dose during the trial

**Characteristics at baseline**			**Initial dose**	**Changes to smoking during follow-up**		**Changes to weight during follow-up**	
IBW	*ABW*	*Smoking status*		*Change to smoking*	*Dose change*	*Change to weight*	*Dose change*
**>60kg**	>60kg	Smoker	bd	Stop Smoking	Reduce to od	Lose ABW<60kg	Reduce to od
>60kg	<60kg	Smoker	od	Stop Smoking	No change	Gain ABW>60kg	Increase to bd
**<60kg**	>60kg	Smoker	od	Stop Smoking	No change	Lose ABW<60kg<br>Gain	No change<br>No change
<60kg	<60kg	Smoker	od	Stop Smoking	No change	Gain	No change
>60kg	>60kg	Non smoker	od	Start smoking	Increase to bd	Lose ABW<60kg<br>Gain	No change<br>No change
>60kg	<60kg	Non smoker	od	Start smoking	No change	Gain	No change
**<60kg**	>60kg	Non smoker	od	Start smoking	No change	Lose ABW<60kg<br>Gain	No change<br>No change
<60kg	<60kg	Non smoker	od	Start smoking	No change	Gain	No change

IBW – Ideal Body Weight; ABW – Actual Body Weight; bd – twice daily; od – once daily

Changes in smoking status:
Non-smoking participants whose ideal body weight (and actual body weight) is more than 60kg who start smoking will have their dose increased to 200mg bd.

Smoking participants whose ideal body weight (and actual body weight) is more than 60kg who cease smoking will have their dose reduced to 200mg od.

To be re-classified as a "smoker" part-way through the study, participants will have smoked for 12 weeks or more. Participants who have given up smoking recently (less than 12 weeks ago) will be classed as a smoker.

To be re-classified as a "non-smoker" part-way through the study, participants must have abstained from smoking for 12 weeks or more. Participants who have given up smoking recently (less than 12 weeks ago) will continue to be classed as a smoker.

At our pre-specified participant contact time-points, we will record information about participants who indicate that they have recently stopped smoking or started smoking. If participants make contact with the study team outwith these time-points to indicate a change in smoking habits, we will again record this information. Both these groups of participants will be contacted again (approximately 12 weeks after they have made the change to their smoking habits) to ascertain whether the change has been sustained to 12 weeks. If it has, the theophylline dose will be changed appropriately.

Changes in weight:
Smoking participants with an ideal body weight of >60kg whose actual body weight falls below 60kg will have their dose reduced to 200mg od.

Smoking participants whose IBW>60kg whose actual body weight increases to above 60kg will have their dose increased to 200mg bd

Information about interacting drugs will be outlined on credit card sized alert card and in more detail on the public areas of the study website.

6.5 PARTICIPANT COMPLIANCE

At each face to face health assessment visit (6 and 12 months) participants will be asked to return empty theophylline/placebo packs. Compliance will be assessed by pill counts of returned study medication.

6.6 OVERDOSE

Theophylline dosages of more than 3g (15 tabs) can be serious in an adult (40 mg/kg in a child). The fatal dose may be as little as 4.5 g (20 tabs) in an adult (60 mg/kg in a child), but is generally higher. With the prolonged release formulations, serious features may develop as long as 12 hours after overdose. Potential symptoms of overdose are listed below:

- Alimentary symptoms: nausea, vomiting (often severe), epigastric pain and haematemesis. If abdominal pain persists pancreatitis should be considered.
- Neurological symptoms: Restlessness, hypertonia, exaggerated limb reflexes and convulsions. Coma may develop in very severe cases.
- Cardiovascular symptoms: Sinus tachycardia is common. Ectopic beats and supraventricular and ventricular tachycardia may follow.
- Metabolic features: Hypokalaemia due to shift of potassium from plasma into cells is common, can develop rapidly and may be severe. Hyperglycaemia, hypomagnesaemia and metabolic acidosis may also occur. Rhabdomyolysis may also occur.

Overdosing will necessitate emergency unblinding and, if necessary, management in accordance with usual clinical practice e.g. www.toxbase.org, UK National Poisons Information Service 0844 892 011.

6.7 OTHER MEDICATIONS

6.7.1 Permitted Medications

Any respiratory medication other than theophylline or roflumilast is permitted. Intervention and control groups will receive usual NHS care: ICS, +/-long acting beta2 agonist (LABA), +/-long acting antimuscarinic.

6.7.2 Prohibited Medications

Drugs not allowed are those known to interact with theophylline and increase serum theophylline.

- antimicrobials: aciclovir, clarithromycin, ciprofloxacin, erythromycin, fluconazole, ketoconazole, levofloxacin, norfloxacin;
- cardiovascular: diltiazem, mexiletine, pentoxifylline, verapamil;
- neurological: bupropion, disulfiram, fluvoxamine, lithium;
- hormonal: medroxyprogesterone, oestrogens;
- immunological: methotrexate, peginterferon alpha, tacrolimus;
- miscellaneous: cimetidine, deferasirox, febuxostat, roflumilast, thiabendazole[29].

In the event that one of these drugs needs to be prescribed for 3 weeks or less, the study medication will be suspended and recommenced after the course of interacting drug has been completed.

In the event that one of these drugs needs to be prescribed for more than 3 weeks, participants will discontinue the study medication (existing supplies returned, no further supplies) but remain in the study and be followed up in accordance with the trial protocol.

7. STUDY ASSESSMENTS

7.1 SAFETY ASSESSMENTS
Safety data will be collected as described in section 10.

7.2 STUDY ASSESSMENTS
After obtaining written informed consent, participants will be assessed as follows:

- At the recruitment visit; face to face
- At approximately 2 weeks; by telephone
- At 6 months; face to face
- At 12 months; face to face
- Post study; examination of hospital and GP records.

In the event that a participant is unable to attend a scheduled assessment visit because of an acute illness e.g exacerbation of COPD, the assessment visit will be appropriately postponed, to be conducted when the participant is stable, ideally within 4 weeks of the scheduled assessment visit. Similarly, patients who are unable to attend a scheduled visit for another reason will have this visit rearranged, ideally within 4 weeks of the scheduled assessment visit. Participants unable to attend for face to face assessment at six and twelve months can be followed up by telephone, a home visit, or sent the questionnaire to compete at home. Similarly, recruitment of patients who, for example have limited mobility or who live some distance from the study site, can be carried out during a home visit.

In addition, we will attempt to contact participants by telephone soon after their study medication has been received.

8. DATA COLLECTION

8.1 SCHEDULE FOR DATA COLLECTION
The schedule for data collection within the study is outlined in Table 6.

Table 6 Schedule of study assessments

Assessment	Recruitment	2 weeks (phone)	Month 6 (face to face)	Month 12 (face to face)	Post study GP records
Assessment of Eligibility Criteria	✓				
Written informed consent	✓				
Demographic data, contact details	✓				
Clinical history	✓				
Drug history	✓		✓	✓	
Smoking status	✓	✓	✓	✓	
Height	✓				
Weight	✓		✓	✓	
Total number COPD exacerbations requiring OCS/antibiotics			✓	✓	✓
Hospital admissions			✓	✓	✓
Health related quality of life	✓		✓	✓	
Disease related health status (CAT, MRC dyspnoea, HARQ)	✓		✓	✓	
Post bronchodilator lung function	✓		✓	✓	
Adverse events/drug reactions		✓	✓	✓	
Health care utilisation	✓		✓	✓	
Patient Compliance			✓	✓	

Potential participants will be informed in the PIL that they can raise any issues with the study team who will arrange further examination if indicated. If the participant or nurse have concerns, these will be discussed with local medically qualified local investigator and suitable arrangements made for the participant to be seen at a later date.

Demographic, clinical data
Demographic, contact, clinical history and clinical examination data will be captured at the recruitment visit.

Drug history
Regular use of prescription drugs will be recorded at recruitment, and the 6 and 12 month assessments.

Smoking history
Smoking history will be recorded at recruitment, and at the 6 and 12 month assessments.

Height & weight
Height will be measured using clinic stadeometer at baseline. Weight will be assessed using scales at recruitment, and the 6 and 12 month assessments.

Number of COPD exacerbations
The primary outcome measure of the total number COPD exacerbations requiring antibiotics/oral corticosteroids whilst on study medication will be ascertained at the 6 and

12 month assessment. Participants will be encouraged to record any exacerbations in the space provided on the outer packaging (carton) used to ship medication, and bring this to their follow-up assessments. The total number of COPD exacerbations will be validated for approximately 20% of participants by examination of GP records post study. Depending on the degree of concordance between patient report and GP records, a decision may be made to check all records. In addition to the 20% of records checked, we will attempt to check GP records for all participants who do not complete the questionnaire and do not attend for follow-up at 12 months.

The ATS/ERS guideline definition of COPD exacerbation will be used: a worsening of patient's dyspnoea, cough and/or sputum beyond day-to-day variability sufficient to warrant a change in management[30]. The minimum management change will be treatment with antibiotics and/or OCS. A minimum of two weeks between consecutive hospitalisations/start of new therapy is necessary to consider events as separate. Severity will be ascertained for each exacerbation.

An operational classification of exacerbation severity will be used:

Level I	Increased use of their SABA
Level II	Use of OCS and/or antibiotics for exacerbation
Level III	Care by services to prevent hospitalisation
Level IV	Admitted to hospital

It can be challenging to differentiate between a COPD exacerbation and pneumonia.

If the patient has been admitted to hospital and a definitive diagnosis is available, we will be able to determine whether this is captured in the data set as an exacerbation, or as a hospital admission (with the diagnosis pneumonia).

If a patient is treated in primary care, a definitive diagnosis may not be available, unless the participant was referred for a chest x-ray. We will capture these cases as COPD exacerbations on the case-report form.

Hospital admissions

The number of unscheduled hospital admissions whilst on study medication will be ascertained by questionnaire at the 6 and 12 month assessments. Emergency admissions consequent upon COPD will also be identified. Participants will be encouraged to record any hospital admissions in the space provided on the outer packaging (carton) used to ship medication, and bring this to their follow-up assessments. The number of hospital admissions will be validated for a sample of participants by examination of GP records post study. Depending on the degree of concordance between patient report and GP records, a decision may be made to check all records. In addition to the sample of records checked, we will attempt to check GP records for all participants who do not complete the questionnaire and do not attend for follow-up at 12 months.

Health related quality of life

Health related quality of life data will be captured at recruitment, and at the 6 and 12 month assessments using EuroQoL 5D (EQ-5D) Index that has been used widely in COPD[31]. EQ-5D was developed as a utility questionnaire and addresses mobility, self-care, usual activities, pain/discomfort and anxiety/depression. The completed instrument can be translated into quality of life utilities suitable for calculation of QALYs through the published UK tariffs.

Disease related health status
Disease related health status will be ascertained at recruitment and at the 6 and 12 month assessments by questionnaire using the COPD Assessment Test (CAT)[32-34]. The CAT is an 8-item unidimensional measure of health status impairment in COPD. The score ranges from 0-40; it correlates very closely with health status measured using the St George Respiratory Questionnaire and is reliable and responsive. The CAT score is preferred since it provides a more comprehensive assessment of the symptomatic impact of COPD.

The MRC dyspnoea scale[1] will also be included in the recruitment, and the 6 and 12 month assessments. This is a single question which assesses breathlessness related to activities.

In selected centres, the HARQ will be used to assess symptoms not elicudated by the CAT or MRC dyspnoea scale. This is a validated self-administered questionnaire which is responsive to treatment effects[40].

Post bronchodilator lung function
Lung function will be measured at recruitment, and 6 and 12 months using spirometry performed to ATS/ERS standards[35]. Spirometry is a routine part of the clinical assessment of people with COPD. Post bronchodilator (LABA within 8 hours, short acting beta2 agonist within 2 hours) FEV1 and FVC will be measured. If necessary lung function will be measured 15 minutes after administration of the participant's own SABA. Widely used predictive equations (ECSC) will be used to compute predicted values for FEV1, FVC[36]. As described in section 4.2, if spirometry is contraindicated, it should not be carried out.

Health care utilisation
Health care utilisation during the previous 6 months will be ascertained at recruitment and the 6 and 12 month assessments (see section 9.3.4).

Adverse reactions and serious adverse events
Adverse reactions and serious adverse events whilst on study medication will be ascertained at the 2 week telephone call and the 6 and 12 month assessments. Serious adverse events/drug reactions will be subject to the serious adverse events reporting protocol (see section 10 for further details).

At the discretion of individual study centres the airways reflux questionnaire (HARQ) will also be used to identify upper GI symptoms at baseline, 6 and 12 months.

Compliance
Compliance with study medication will be assessed at 6 and 12 month assessments. Participants will be asked to return empty drug bottles. Compliance will be calculated by pill counting.

Mortality
Deaths during the follow-up period will be reported as SAEs.

8.2 SOURCE DATA
Our primary outcome is exacerbations of COPD necessitating changes in management as reported by the participant. For many of our secondary outcomes (total number of COPD admissions requiring hospital admission, episodes of pneumonia, emergency hospital admissions (all causes), serious adverse events, adverse reactions, total dose of inhaled corticosteroid, utilisation of primary or secondary health care for respiratory events) the

primary source will also be patient-report, although additional details may be available from other sources (including medical records). The health status outcomes are patient reported.

At baseline, study data is collected on hard copy case report form. This is considered to be the source document.

At follow-up (each telephone contact, and follow-up at 6 and 12 months), study data can be collected on hard copy case report form or entered directly into the study website.

- If hard copy case report forms are completed, these are considered to be the source document. These will then entered by the local study team onto the study website.
- If the data is entered directly into the study website, the electronic record is considered to be the source document. In order to maintain a copy of the data that is independent from the sponsor copy, sites will be encouraged to print or save a copy of the electronic data. The study website will provide this facility.

Each website user will have their own user account and password. These must not be shared. The study website has a full audit trail and every data entry made (or changed) is logged to the specific user.

For all case report forms, there is an electronic record (as part of the study website) which indicates whether the case report form was completed on line (no paper copy) or not. This will allow identification of the source document.

Participants will complete questionnaires at baseline and at 6 and 12 month follow-up. The hard copy of these questionnaires will be considered the source document.

9. STATISTICS, DATA ANALYSIS AND ECONOMIC EVALUATION

9.1 SAMPLE SIZE CALCULATION

The multicentre Evaluation of COPD Longitudinally to Identify Predictive Surrogate Endpoints (ECLIPSE)[38] study reported the frequency of COPD exacerbation in 2138 patients. For patients identical to our target population (≥2 self reported COPD exacerbations in a year requiring antibiotics and/or OCS), the mean (SD) number of COPD exacerbations within one year was 2.22 (1.86), (Dr Nick Locantore, ECLIPSE statistician, personal communication). Assuming a similar rate in the placebo arm we need 669 subjects in each arm of the trial to detect a clinically important reduction in COPD exacerbations of 15% (i.e. from an average of 2.22 to 1.89) with 90% power at the two-sided 5% significance level. Allowing for an estimated 6% loss to follow-up[26] we will therefore aim to recruit 712 subjects per arm (i.e. 1424 in total, an average of 203 in each of the 7 centres).

9.2 PROPOSED ANALYSES

Statistical analyses will be according to the intention to treat principle with a per protocol analysis performed as a sensitivity. The per protocol analysis will exclude participants who were not compliant (at less than 75%) with their study medication. All analyses will be governed by this comprehensive SAP which will be agreed by the Trial Steering Committee (TSC) and approved by the independent Data Monitoring Committee (DMC) prior to any analyses being undertaken. Unless pre-specified, a 5% two-sided significance level will be used to denote statistical significance throughout. There will be no interim analyses undertaken. In line with other recent COPD trials it is not envisaged that the DMC will be monitoring the study in order to terminate it prematurely on finding evidence of

overwhelming benefit – nor will they terminate it for futility.

The primary outcome - number of COPD exacerbations requiring antibiotics and/or oral corticosteroids in the 12 months after randomisation - will be compared between randomised groups using negative binomial regression (as an alternative to the usual Poisson approach) with length of time in the study as an offset. Estimates will then be adjusted for centre and other baseline covariates that are known to be strongly related to outcome (e.g. age, smoking, COPD hospitalisations in year prior to study – these will be pre-specified in the SAP). Despite the patients being followed-up for a single year, some will withdraw early and, given their chronic illness, some will inevitably die. Use of an overdispersion parameter should improve the model estimates as it takes between patient variability in exacerbation rate into account. The negative binomial model assumes that each patient has their own underlying exacerbation rate which follows a Poisson distribution, but the expected rate is allowed to vary across patients according to a gamma distribution. The shape parameter from the gamma distribution explicitly represents the variability between patients. The negative binomial model coincides with the Poisson model when this dispersion parameter is zero. Assumptions of this model are that exacerbation counts are assumed to vary about means that differ for each patient, with means varying across the population. Hence, the negative binomial model more effectively accounts for increased exacerbation events amongst patients withdrawing early.

To further assess the impact of death (estimated at around 6% of the randomised subjects in the first 12 months after randomisation) on our potential treatment effect, we will undertake a sensitivity analysis by excluding those subjects who have died. If there is any indication of a differential effect on deaths by treatment, we may consider models that allow the censoring to be informative. For participants that are lost to follow up at some time during the 12 month follow-up (estimated to be around 6% of those randomised), their information will be included in the statistical models up to the point that they are lost to follow up. We will undertake sensitivity analyses using multiple imputation (assuming data are missing at random), and, if necessary, and the data permit, specify the mechanism of missing data via a pattern mixture model assuming informative missingness.

The secondary outcomes - total number of COPD exacerbations requiring hospital admission and total number of emergency hospital admissions (all causes) - will each be analysed in the same way as for the primary outcome described above.

Disease related health status (measured using the COPD Assessment Test (CAT)), generic health-related quality of life (EQ5D) and Hull Airways Reflux Questionnaire (HARQ), FEV1 and FVC are each measured at baseline, and at 6 and 12 month follow-up. A mixed effects model will be used to compare each outcome by randomisation group unadjusted and adjusted for centre, patient characteristics and/or baseline clinical variables. Mixed effects models assess rates of change with allowance for the correlation structure of the repeated measures data. Fixed effects will include assessment, treatment, centre and other relevant patient related variables and patient will be fitted as a random effect. A treatment-time interaction will be included to assess the differential treatment effect on rate of change in outcome. Alternative correlation structures will be considered and the most appropriate selected based on the log-likelihood and Akaike's Information Criterion (AIC) of the model. All randomised patients with at least one valid measurement will be included in the analysis and missing outcome data assumed to be missing at random.

As sensitivity analyses, (a) the mixed effects models will be repeated on those patients who

survive the 12 month follow-up only (b) where appropriate to do so a pre-specified value will be imputed for those patients who died during follow-up (e.g. zero value for EQ5D) (c) we will undertake multiple imputation assuming data are missing at random (d) if missing data are thought to be informative, then we will consider the use of appropriate models such as the pattern mixture model.

Descriptive statistics for health care utilisation and the occurrence of adverse events will be produced and, if appropriate, a comparison will be made between randomised groups using the chi-squared test, t-test etc. Poisson regression may be used to compare multiple events of the same type between randomisation groups. All-cause mortality rate will be compared between randomised group using a log-rank test and Kaplan-Meier survival curves. Adjustment for potential covariates will be undertaken using Cox proportional hazards regression.

Compliance will be assessed by pill counting of the returned containers at each of the follow up visits and the proportion of compliant patients compared between randomisation groups using the chi-squared test.

9.3 ECONOMIC EVALUATION

An NHS economic evaluation will be conducted in two stages. Firstly, the cost-effectiveness of treatment will be calculated for the within trial period based on observed data. Secondly, the results of the trial will be extrapolated to patient lifetimes using cost-effectiveness modelling.

The within trial analysis will make use of the health care resource use data (translated to a cost-per-patient using unit costs standard reference sources), the exacerbation rate associated with the treatment arms, and the quality of life effects estimated from the EQ-5D combined with utility data to calculate QALYs. Non-parametric bootstrapping will be used to capture sampling uncertainty in the observed data and results will be presented as cost-per-exacerbation avoided and cost-per-QALY gained within the trial period with accompanying confidence intervals (or cost-effectiveness acceptability curves if more appropriate).

The extrapolation analysis will make use of regression estimates of exacerbation on cost and quality of life from the trial, as well as previously published models of COPD, to guide the extrapolation to patient lifetimes. In addition to sampling uncertainty, extensive sensitivity, analysis will be performed to understand the importance of alternative modelling assumptions for the extrapolated results.

9.3.2 Collection of data

Data collection from the trial will focus on estimating the use of primary or secondary NHS health care resources.

9.3.3 Participant Costs

Direct health care costs falling on the participant costs will not be included. We do not believe that these costs will be substantial and in any case fall outside of the health service reference case for this analysis.

9.3.4 NHS Costs of Health Services Used

Health care utilisation during the previous 6 months will be ascertained at recruitment and the 6 and 12 month assessments by a modified version of the The Client Service Receipt

Inventory (CSRI[37]). The CSRI is a research questionnaire for retrospectively collecting cost-related information about participants use of health and social care services.

9.3.5 Cost Effectiveness

Following a method we have previously used in large international trial for COPD treatments[39], the within trial analysis will use regression techniques to simultaneously capture heterogeneity of cost-effectiveness among the patients studied, while adjusting for any missing data.

10. ADVERSE EVENTS

The Investigator is responsible for the detection and documentation of events meeting the criteria and definitions detailed below.

Full details of contraindications and side effects that have been reported following administration of the trial drug can be found in the relevant Summary of Product Characteristics (SmPC) in Appendix 2.

Participants should be instructed to contact their Investigator at any time after consenting to join the trial if any new or untoward symptoms develop. The Investigator should initiate the appropriate treatment according to their medical judgment. Participants with SAEs or ARs present at the last visit will be followed up, if appropriate, until resolution of the event.

10.1 DEFINITIONS

An adverse event (AE) is any untoward medical event affecting a clinical trial participant. Each initial AE will be considered for severity, causality or expectedness and may be reclassified as a serious event or reaction based on prevailing circumstances.

An adverse reaction (AR) is where it is suspected that an AE has been caused by a reaction to a trial drug.

A serious adverse event (SAE), serious adverse reaction (SAR) or suspected unexpected serious adverse reaction (SUSAR) is any AE, AR or UAR that at any dose:
results in death;

- is life threatening (i.e. the subject was at risk of death at the time of the event; it does not refer to an event which hypothetically might have caused death if it were more severe)*;
- requires hospitalisation*,
- results in persistent or significant disability or incapacity;
- is a congenital anomaly or birth defect.

**Notes on definitions:*
In this trial, hospitalisations for treatment planned prior to randomisation and hospitalisation for elective treatment of a pre-existing condition will not be considered or recorded or reported as an SAE. Complications occurring during such hospitalisation will also not be considered, recorded or reported as an SAE – unless there is a possibility that the complication arose because of the study medication (ie a possible adverse reaction).

In this trial, exacerbations of COPD, pneumonia or hospital admissions as a consequence of exacerbations of COPD or pneumonia will not be considered as AEs or SAEs because they are

primary and secondary outcomes for the trial. These will be recorded as part of the trial outcomes, but will not be considered or recorded or reported as AEs or SAEs.

10.2 DETECTING ARs AND SAEs

Theophylline has been used for 70 years for the treatment of asthma and COPD, in this trial theophylline is being administered in accordance with its license.

SAEs will be recorded from the time a participant consents to join the study until the last study visit (see notes in section 10.1). All adverse reactions will be recorded.

The researcher should ask about the occurrence of SAEs, ARs and SARs at each follow-up visit. Open-ended and non-leading verbal questioning of the participant should be used to enquire about SAE, AR and SAR occurrence. Participants should also be asked if they have been admitted to hospital, used any new medicines or changed concomitant medication regimens. If there is any doubt as to whether a clinical observation is an AR, the event should be recorded.

For patients who do not attend for follow-up, a supplementary questionnaire will be sent to them at 6 and 12 months which will ask about the occurrence of SAEs, ARs and SARs.

10.3 RECORDING SAEs

Depending on severity, when an SAE occurs, it is the responsibility of the Investigator to review all documentation (e.g. hospital notes, laboratory and diagnostic reports) related to the event. The Investigator should then record all relevant information on the SAE form. Information to be collected includes dose, type of event, onset date, Investigator assessment of severity and causality, date of resolution, as well as treatment required, investigations needed and outcome.

10.4 EVALUATION OF SAEs

Seriousness, causality, severity and expectedness should be evaluated as though the participant is taking active drug. Cases that are considered serious, possibly, probably or definitely related to drug and unexpected (i.e. SUSARs) are likely to be unblinded.

10.4.1 Assessment of Seriousness

The Investigator should make an assessment of seriousness as defined in Section 10.1.

10.4.2 Assessment of Causality

The Investigator must make an assessment of whether the SAE is likely to be related to treatment according to the following definitions:

- Unrelated: where an event is not considered to be related to the study drug.
- Possibly: although a relationship to the study drug cannot be completely ruled out, the nature of the event, the underlying disease, concomitant medication or temporal relationship make other explanations possible.
- Probably: the temporal relationship and absence of a more likely explanation suggest the event could be related to the study drug.
- Definitely: The known effects of the study drug or its therapeutic class, or based on challenge testing, suggest that study drug is the most likely cause.

All AEs or SAEs judged as having a reasonable suspected causal relationship (e.g. possibly, probably, definitely related) to the study drug will be considered as ARs or SARs. All AEs or SAEs judged as being related (e.g. possibly, probably, definitely) to an interaction between

the study drug and another drug will also be considered to be ARs or SARs.

Alternative causes such as natural history of the underlying disease, concomitant therapy, other risk factors and the temporal relationship of the event to the treatment should be considered. The blind should not be broken for the purpose of making this assessment.

10.4.3 Assessment of Severity

The Investigator should make an assessment of severity for each SAE and record this on the CRF according to one of the following categories:

- Mild: an event that is easily tolerated by the participant, causing minimal discomfort and not interfering with every day activities.
- Moderate: an event that is sufficiently discomforting to interfere with normal everyday activities.
- Severe: an event that prevents normal everyday activities.

Note: the term ‘severe’, used to describe the intensity, should not be confused with ‘serious’ which is a regulatory definition based on participant/event outcome or action criteria (section 10.1). For example, a headache may be severe but not serious, while a minor stroke is serious but may not be severe.

10.4.4 Assessment of Expectedness

If an event is judged to be an AR/SAR, the evaluation of expectedness should be made based on knowledge of the reaction and the relevant product information documented in the SmPC.

In summary, the expected side effects of theophylline are:

Immunological	Anaphylactic, anaphylactoid reactions
Metabolic	Hyperuricaemia
Psychiatric	Agitation, anxiety, insomnia
CNS	Convulsions, dizziness, headache, tremor
Cardiac	Atrial/sinus tachycardia, palpitations,
Gastrointestinal	Abdominal pain, diarrhoea, gastric irritation, gastro-oesophageal reflux, nausea, vomiting
Skin	Pruritus, rash
Renal and urinary	Diuresis, urinary retention

Patients with COPD are at increased risk of death (for example from cancer), cardiovascular (for example myocardial infarction, heart failure, angina) and cerebrovascular events (for example stroke, transient ischaemic attack).

10.5 REPORTING OF SAEs / SARs / SUSARs

Once the Investigator becomes aware that an SAE has occurred in a study participant, they must report the information to the Trial Office within 24 hours of becoming aware of the event, the Trial Office will report to the Sponsor within 24 hours of becoming aware of the event as per the current UoA-NHSG-SOP-014. The SAE form must be completed as thoroughly as possible with all available details of the event, and signed by the Investigator

or designee. If all the required information is not available at the time of reporting, the Investigator must ensure that any missing information is provided as soon as this becomes available. It should be indicated on the report that this information is follow-up information of a previously reported event.

10.6 REGULATORY REPORTING REQUIREMENTS

The Chief Investigator is responsible for informing the MHRA and the main REC of relevant safety issues. Fatal or life threatening SUSARs will be reported to MHRA no later than **7 calendar days** and all other SUSARs will be reported no later than **15 calendar days** after they are first aware of the reaction.

An annual Development Safety Update Report (DSUR) will be submitted to the MHRA and the main REC listing all SARs and SUSARs. The Chief Investigator is responsible for submitting annual DSURs to the MHRA and the main REC on the anniversary of the Clinical Trial Authorisation approval.

10.7 FOLLOW UP PROCEDURES

After initially recording and reporting an SAE, the Investigator is required to follow each participant as indicated by clinical practice. Follow up information on an SAE should be reported to the Sponsor as per SOP UoA-NHSG-SOP-014.

11. PREGNANCY

Although risk benefit considerations indicate that theophylline is safe to use in pregnancy[29], the SMPC indicates that theophylline should not be administered in pregnancy unless clearly necessary, and should only be given to breast feeding women when the anticipated benefits outweigh the risk to the child. Current (or planned pregnancy during the study) or current breastfeeding are exclusion criteria. Pregnancy is not considered an SAE however the investigator must collect pregnancy information for female trial subjects. The Investigator should record the information on a Pregnancy Notification Form and submit this to the Trial Office within 14 days of being made aware of the pregnancy. In such cases, trial medication should be ceased. Any pregnancy that occurs in a trial subject during a trial should be followed to outcome. In some circumstances, it may be necessary to monitor the development of the newborn for an appropriate period post delivery. Should the trial subject not wish for the pregnancy to followed to outcome or beyond, this should be noted in the CRF and medical notes as appropriate.

There is no evidence of teratogenic potential in men. Therefore, we do not propose to collect data on pregnancies in the female partners of male participants, or to follow any such pregnancies up.

12. TRIAL MANAGEMENT AND OVERSIGHT ARRANGEMENTS

12.1 PROJECT MANAGEMENT GROUP

The trial will be coordinated by a Project Management Group, consisting of the grantholders (Chief Investigator and other grant holders as appropriate), the Trial Manager and other senior members of the Trials Unit.

12.2 TRIAL MANAGEMENT

A Trial Manager will oversee the study and will be accountable to the Chief Investigator.

The Trial Manager will be based in a Central Trial Office which will provide support to each site.

12.3 TRIAL STEERING COMMITTEE
A Trial Steering Committee (TSC), with independent members, will be established to oversee the conduct and progress of the trial. The terms of reference of the Trial Steering Committee, the template for reporting and the names and contact details of members of the TSC will be filed in the Trial Master File.

12.4 DATA MONITORING COMMITTEE
An independent Data Monitoring Committee (DMC) will be established to oversee the safety of subjects in the trial. The terms of reference of the DMC and the names and contact details will be filed in the Trial Master File.

12.5 INSPECTION OF RECORDS
Principal Investigators and institutions involved in the study will permit trial related monitoring, audits, REC review, and regulatory inspection(s). In the event of an audit, the Investigator agrees to allow the Sponsor, representatives of the Sponsor or regulatory authorities direct access to all study records and source documentation.

12.6 STUDY MONITORING
A monitor, designated by the Sponsor, or an appointed local monitor will visit the Aberdeen study site prior to the start of the study, during the course of the study and will undertake a close down visit at study end. The Trial Office will carry out remote central monitoring of accumulating data and consent forms from all sites. This may trigger on-site monitoring at any of the study sites. In addition, the Trial Office will facilitate monitoring by local R&D departments at each of the sties, should this be requested.

A copy of the monitoring plan will be filed in the Trial Master File.

13. GOOD CLINICAL PRACTICE

13.1 ETHICAL CONDUCT OF THE STUDY
The study will be conducted in accordance with the principles of Good Clinical Practice (GCP).

A favourable ethical opinion will be obtained from the appropriate REC and local NHS R&D approval will be obtained prior to commencement of the study.

13.2 REGULATORY COMPLIANCE OF THE STUDY
The study will not commence until a Clinical Trial Authorisation (CTA) is obtained from the appropriate Regulatory Authority. The protocol and study conduct will comply with the Medicines for Human Use (Clinical Trials) Regulations 2004, and any relevant amendments.

13.3 INVESTIGATOR RESPONSIBILITIES
The Investigator is responsible for the overall conduct of the study at the site and compliance with the protocol and any protocol amendments. In accordance with the principles of GCP, the following areas listed in this section are also the responsibility of the Investigator. Responsibilities may be delegated to an appropriate member of study site staff.

A Delegation Log will be prepared for each site, detailing the delegated responsibilities of each member of staff working on the trial. This should be signed by those named on the list.

13.3.1 Informed Consent

The Investigator is responsible for ensuring informed consent is obtained before any protocol specific procedures are carried out. The decision of a participant to participate in clinical research is voluntary and should be based on a clear understanding of what is involved.

Participants must receive adequate oral and written information – appropriate Participant Information and Informed Consent Forms will be provided. The oral explanation to the participant should be performed by the Investigator or designated person, and must cover all the elements specified in the Participant Information Sheet/Informed Consent.

The participant must be given every opportunity to clarify any points they do not understand and, if necessary, ask for more information. The participant must be given sufficient time to consider the information provided. It should be emphasised that the participant may withdraw their consent to participate at any time without loss of benefits to which they otherwise would be entitled.

The participant should be informed and agree to their medical records being inspected by regulatory authorities but understand that inspection is undertaken by authorised personnel and their data will remain confidential.

The Investigator or delegated member of the trial team and the participant should sign and date the Informed Consent Form(s) to confirm that consent has been obtained. The participant should receive a copy of this document and a copy should be filed in the participant's medical records (for patients recruited in primary care, this will be in their GP notes rather than their hospital medical records). Copies should also be filed in the Trial Master File (TMF) and the Investigator Site File (ISF).

13.3.2 Study Site Staff

The Investigator must be familiar with the IMP, protocol and the study requirements. It is the Investigator's responsibility to ensure that all staff assisting with the study are adequately informed about the IMP, protocol and their trial related duties.

13.3.3 Data Recording

The Investigator is responsible for the quality of the data recorded in the CRF.

13.3.4 Investigator Documentation

Prior to beginning the study, each Investigator will be asked to provide particular essential documents to the Sponsor, including but not limited to:

- An original signed Investigator's Declaration (as part of the Clinical Trial Agreement documents)

The Chief Investigator, with the agreement of the Sponsor, will ensure all other documents required for compliance with the principles of GCP are retained in a TMF and that appropriate documentation is available in local ISFs.

13.3.5 GCP Training

Study staff should be qualified, by education, training and experience, appropriate to their role in the project.

13.3.6 Confidentiality

All evaluation forms, reports, and other records must be identified in a manner designed to maintain participant confidentiality. All records must be kept in a secure storage area with limited access.

Clinical information will not be released without the written permission of the participant, except as necessary for monitoring and auditing by the Sponsor, its designee, Regulatory Authorities, or the REC.

The Investigator and study site staff involved with this study may not disclose or use for any purpose other than performance of the study, any data, record, or other unpublished, confidential information disclosed to those individuals for the purpose of the study. Prior written agreement from the Sponsor or its designee must be obtained for the disclosure of any said confidential information to other parties.

13.3.7 Data Protection

All Investigators and study site staff involved with this study must comply with the requirements of the Data Protection Act 1998 with regard to the collection, storage, processing and disclosure of personal information and will uphold the Act's core principles. Access to collated participant data will be restricted to those clinicians treating the participants.

Computers used to collate the data will have limited access measures via user names and passwords.

Published results will not contain any personal data that could allow identification of individual participants.

14. STUDY CONDUCT RESPONSIBILITIES

14.1 PROTOCOL AMENDMENTS

Any changes in research activity, except those necessary to remove an apparent, immediate hazard to the participant, must be reviewed and approved by the Sponsor. Amendments to the protocol must be submitted in writing to the appropriate REC, Regulatory Authority and local R&D for approval prior to participants being enrolled into an amended protocol.

Amendments to other documentation should not be implemented until appropriate approvals are in place.

14.2 PROTOCOL VIOLATIONS AND DEVIATIONS

The Investigator should not implement any deviation from the protocol without agreement from the Chief Investigator and appropriate REC, Regulatory Authority and R&D approval except where necessary to eliminate an immediate hazard to trial participants.

In the event that an Investigator needs to deviate from the protocol, the nature of and reasons for the deviation should be recorded in the CRF. If this necessitates a subsequent protocol amendment, this should be submitted to the REC, Regulatory Authority and local

R&D for review and approval if appropriate.

14.3 STUDY RECORD RETENTION

All study documentation will be kept for at least 15 years after publication of the study data. Copies of consent forms will be forwarded to Aberdeen on a regular basis. At the end of each particiapnt's follow-up, case report forms and questionnaires will be returned for archiving in Aberdeen. The site files will be archived at each site.

14.4 END OF STUDY

The end of clinical follow-up for each participant is defined as completion of the follow-up visit at 12 months. The end of clinical follow-up is when the last participant completes their follow-up visit at 12 months. The end of the trial is defined as the end of funding.

The Investigators and/or the Trial Steering Committee have the right at any time to terminate the study for clinical or administrative reasons.

The end of the trial will be reported to the REC and Regulatory Authority within 90 days, or 15 days if the study is terminated prematurely. The Investigators will inform participants and ensure that the appropriate follow up is arranged for all involved.

A summary report of the trial will be provided to the REC and Regulatory Authority within 1 year of the end of the trial. An end of trial report should also be issued to the funders at the end of funding.

14.5 CONTINUATION OF DRUG FOLLOWING THE END OF STUDY

We do not plan to unblind individual participants at the end of their clinical follow-up. Thus, participants will not be told whether they were taking low dose theophylline or placebo. However, if a participant wishes to take low dose theophylline at their clinical follow-up, the GP can be advised of this by letter. In the letter we will indicate to the GP that the patient may have been on placebo or low-dose theophylline. If the GP considers this appropriate, they can prescribe theophylline if it is available on the local NHS formulary.

15. REPORTING, PUBLICATIONS AND NOTIFICATION OF RESULTS

15.1 AUTHORSHIP POLICY

Ownership of the data arising from this study resides with the study team. On completion of the study, the study data will be analysed and tabulated, and a clinical study report will be prepared. The publication policy is included in Appendix 3.

15.2 PUBLICATION

The clinical study report will be used for publication and presentation at scientific meetings. Investigators have the right to publish orally or in writing the results of the study. Summaries of results will also be made available to Investigators.

A summary of the study findings will be sent to surviving trial participants (unless they request otherwise).

15.3 PEER REVIEW

All reports of work arising from the TWICS trial including conference abstracts should be peer reviewed by the Project Management Group prior to submission – for further details see Appendix 3.

REFERENCES

1. Chronic obstructive pulmonary disease: Management of chronic obstructive pulmonary disease in adults in primary and secondary care (partial update) . Clinical Guideline CG101. London; National Institute for Health & Clinical Excellence, 2010. Available at: http://publications.nice.org.uk/chronic-obstructive-pulmonary-disease-cg101. Accessed April, 2012.

2. Clearing the air: a national study of chronic obstructive pulmonary disease. London; Commission for Healthcare Audit & Inspection, 2006. Available at: http://archive.cqc.org.uk/_db/_documents/COPD_report1_200607272728.pdf. Accessed April, 2012.

3. Britton M. The burden of COPD in the U.K.: results from the Confronting COPD survey. Respir Med 2003;97:S71-9.

4. Donaldson GC, et al. Relationship between exacerbation frequency and lung function decline in chronic obstructive pulmonary disease. Thorax 2002;57:847-52.

5. Donaldson GC, et al. Exacerbations and time spent outdoors in chronic obstructive pulmonary disease. Am J Resp Crit Care Med 2005;171:446-52.

6. Seemungal TA, et al. Effect of exacerbation on quality of life in patients with chronic obstructive pulmonary disease. Am J Resp Crit Care Med 1998;157:1418-22.

7. Soler-Cataluna JJ, et al. Severe acute exacerbations and mortality in patients with chronic obstructive pulmonary disease. Thorax 2005;60:925-31.

8. GINA Report, Global Strategy for Asthma Management and Prevention. Global Iniatiative for Asthma, 2011. Available at: http://www.ginasthma.org/guidelines-gina-report-global-strategy-for-asthma.html. Accessed April, 2012.

9.Calverley PM, et al. Salmeterol and fluticasone propionate and survival in chronic obstructive pulmonary disease. N Engl J Med 2007;356:775-89.

10. Barnes PJ, et al. Corticosteroid resistance in chronic obstructive pulmonary disease: inactivation of histone deacetylase. Lancet 2004;363:731-3.

11. Culpitt SV, et al. Effect of high dose inhaled steroid on cells, cytokines, and proteases in induced sputum in chronic obstructive pulmonary disease. Am J Resp Crit Care Med 1999;160:1635-9.

12.Culpitt SV, et al. Impaired inhibition by dexamethasone of cytokine release by alveolar macrophages from patients with chronic obstructive pulmonary disease. Am J Resp Crit Care Med 2003;167:24-31.

13. Hattotuwa KL, et al. The effects of inhaled fluticasone on airway inflammation in chronic obstructive pulmonary disease: a double-blind, placebo-controlled biopsy study. Am J Resp Crit Care Med 2002;165:1592-6.

14. Ito K, et al. Glucocorticoid receptor recruitment of histone deacetylase 2 inhibits interleukin-1beta-induced histone H4 acetylation on lysines 8 and 12. Mol Cell Biol 2000;20:6891-903.

15. Ito K, et al. Decreased histone deacetylase activity in chronic obstructive pulmonary disease. N Engl J Med 2005;352:1967-76.

16. Ito K, et al. Histone deacetylase 2-mediated deacetylation of the glucocorticoid receptor enables NF-kappaB suppression. J Exp Med 2006;203:7-13.

17. Barnes PJ. Targeting the epigenome in the treatment of asthma and chronic obstructive pulmonary disease. Proc Am Thorac Soc 2009;6:693-6.

18. Marwick JA, et al. Inhibition of PI3Kdelta restores glucocorticoid function in smoking-induced airway inflammation in mice. Am J Resp Crit Care Med 2009;179:542-8.

19. Ito K, et al. A molecular mechanism of action of theophylline: Induction of histone deacetylase activity to decrease inflammatory gene expression. Proc Natl Acad Sci U S A 2002;99:8921-6.

20. Cosio BG, et al. Theophylline restores histone deacetylase activity and steroid responses in COPD macrophages. J Exp Med 2004;200:689-95.

21. Cosio BG, et al. Low-dose theophylline enhances the anti-inflammatory effects of steroids during exacerbations of COPD. Thorax 2009;64:424-9.

22. Ford PA, et al. Treatment effects of low-dose theophylline combined with an inhaled corticosteroid in COPD. Chest 2010;137:1338-44.

23. Cyr MC, et al. Effect of theophylline on the rate of moderate to severe exacerbations among patients with chronic obstructive pulmonary disease. Br J Clin Pharmacol 2008;65:40-50.

24. Barr RG, et al. Methylxanthines for exacerbations of chronic obstructive pulmonary disease: meta-analysis of randomised trials. BMJ 2003;327:643.

25. Powell JR, et al. Theophylline disposition in acutely ill hospitalized patients. The effect of smoking, heart failure, severe airway obstruction, and pneumonia. Am Rev Respir Dis 1978;118:229-38.

26. Ram FS, et al. Oral theophylline for chronic obstructive pulmonary disease. Cochrane Database of Systematic Reviews 2002, Issue 3. Art. No.: CD003902. DOI: 10.1002/14651858.CD003902.

27. Makino S, et al. A prospective survey on safety of sustained-release theophylline in treatment of asthma and COPD. Allergol Int 2006;55:395-402.

28. Ohta K, et al. A prospective clinical study of theophylline safety in 3810 elderly with asthma or COPD. Respir Med 2004;98:1016-24.

29. British National Formulary No 63. London: BMJ Group & Royal Pharmaceutical Society, 2012. Available at: http://www.medicinescomplete.com/mc/bnf/current/. Accessed April, 2012.

30. Celli BR, MacNee W, ATS/ERS Task Force. Standards for the diagnosis and treatment of patients with COPD: a summary of the ATS/ERS position paper. Eur Resp J 2004;23:932-46.

31. The EuroQol Group. EQ-5D. Rotterdam:EuroQol, 2012. Available at: http://www.euroqol.org/. Accessed April, 2012.

32. COPD Assessment Test. Brentford, UK:GlaxoSmithKline, 2009. Available at: http://www.catestonline.co.uk/. Accessed April, 2012.

33. Dodd JW, et al. The COPD assessment test (CAT): response to pulmonary rehabilitation. A multicentre, prospective study. Thorax 2011;66:425-9.

34. Jones PW, et al. Development and first validation of the COPD Assessment Test. Eur Resp J 2009;34:648-54.

35. Miller MR et al. Standardisation of spirometry. Eur Resp J 2005;26:319-38.

36. Quanjer PH, et al. Lung volumes and forced ventilatory flows. Report Working Party Standardization of Lung Function Tests, European Community for Steel and Coal. Official Statement of the European Respiratory Society. Eur Resp J Suppl 1993;16:5-40.

37. Barrett B, Byford S. Collecting service use data for economic evaluation in DSPD populations. Br J Psychiatry, 2007, 190 (sup 49), s75-s78.

38. Hurst JR et al. Evaluation of COPD Longitudinally to Identify Predictive,Surrogate Endpoints. Susceptibility to exacerbation in chronic obstructive pulmonary disease. N Engl J Med 2010;363:1128-38.

39. Briggs AH, et al. Is treatment with ICS and LABA cost effective for COPD? Multinational economic analysis of the TORCH study. Eur Respir J 2010; 35: 532-9.

40. Morice AH, Faruqi S, Wright CE, Thompson R, Bland JM: Cough hypersensitivity syndrome: a distinct clinical entity. Lung 2011, 189:73-79.

APPENDIX 1: Rationale for the low dose theophylline strategy

Population theophylline pharmacokinetic studies, during the 1970s and 80s have demonstrated the link between disease status, weight and that smoking decreases the half life of theophylline and increases its clearance [1-7]. It has been shown that COPD patients that do not smoke have a reduced clearance compared to healthy volunteers [4]. Based on the above data and our publications[8-11] then an average population clearance value of theophylline in a non smoker is 40ml/hr/kg which is reduced to 32ml/hr/kg in a subject with COPD and by a further 20% if they have other related disease (eg severe congestive heart failure). This corresponds to the fast, normal and slow categories of serum theophylline pharmacokinetic modelling, for COPD patients, provided in the table below. Smoking induces the theophylline clearance by approximately 60% which gradually returns to normal levels when they stop smoking. Other relevant population pharmacokinetic data, that is useful for loading doses, is a volume of distribution of 0.5L/kg [1-11].

The BNF recommends an aminophylline infusion of 0.5mg/kg/hr. Based on a clearance of 40ml/hr/kg and the following pharmacokinetic model

$$\mathrm{Css} = \frac{\mathrm{F} \times \mathrm{D}}{\mathrm{Cl} \times \tau}.$$

[Where Css is the steady state theophylline concentration, F is the bioavailability of theophylline (F=0.8 for aminophylline preparations), D is the dose, Cl is the clearance (using a clearance of 40ml/hr/Kg) and τ is the dosage interval (1 hour for an intravenous infusion)] the predicted Css would be:

$$\mathrm{Css} = \frac{0.8 \times 0.5\mathrm{mg/hr/kg}}{0.04\mathrm{L/hr/kg} \times 1}.$$
$$= 10\mathrm{mg/L}$$

Note that the predicted Css is irrespective of weight (see above equation).
The predicted Css in a COPD non smoker classified with a slow, normal and fast theophylline clearance given an infusion of 0.5 mg/hr/kg would be 10. 12.5 and 16.7 mg/L. In a smoker the respective predicted Css be 6.3, 7.8 and 10.4 mg/L.

The pharmacokinetic model for a loading dose is

$$\mathrm{Co} = \frac{\mathrm{F} \times \mathrm{D}}{\mathrm{V}}$$

Where Co is the concentration immediately after the slow intravenous bolus dose of aminophylline, F is the bioavailability (F=0.8 for aminophylline) and V is the volume of distribution. A loading dose of 5mg/kg would provide a Co of

$$\mathrm{Co} = \frac{0.8 \times 5\mathrm{mg/kg}}{0.5\,\mathrm{L/kg}}$$
$$= 8\mathrm{mg/L}$$

Therefore a loading dose of aminophylline in COPD patients weighing 40-100kg would provide a Co ranging from 10 to 4 mg/L. There is a linear relationship between Css and weight. Similarly if the loading dose was 500mg aminophylline then the predicted Css would be double that for the 250mg dose.

The use of actual weight or ideal body weight has been shown to have an effect on the clearance of theophylline in young adults that smoke. If a patient is obese they may be given a high dose when their actual weight is used. It is good practice to assume this occurs in all patients and thus use ideal body weight. Ideal body weight can be calculated using the following equations
IBWfemale =45+ (2.3 x each inch above 60) kg [45 + 0.9(height in cms-152) Kg]
IBWmale = 50 + (2.3 x each inch above 60) kg [50 + 0.9(height in cms-152) Kg]

The ideal body weight is used unless the actual weight is lower than the ideal body weight. For oral theophylline dosing the pharmacokinetic model is

$$\mathrm{Css} = \frac{\mathrm{F} \times \mathrm{D}}{\mathrm{Cl} \times \tau}$$

Where Css is the steady state theophylline concentration, F is the bioavailability of theophylline (F=1 for theophylline preparations), D is the dose, Cl is the clearance and τ is the dosage interval (either 12 or 24 hours). Using this model and the population theophylline clearance values for COPD patients, in smokers and non smokers, described above then predicted Css are a follows.

Table 1 The results of pharmacokinetic modelling for theophylline doses 200mg bd and od for current smoking/not- current smoking subjects by weight and theophylline clearance. The serum theophylline concentrations using the dosing schedule are shaded.(Prof Henry Chrystyn, personal communication)

		Theophylline 200mg bd				Theophylline 200mg od		
		Steady state (Css) serum theophylline concentration (mg/l)						
	Ideal body weight (kg)	Subject theophylline clearance				Subject theophylline clearance		
		Slow	Normal	Fast		Slow	Normal	Fast
Not current Smoker	40.1-50	17.4	13.0	10.4		8.7	6.5	5.2
	50.1-60	13.9	10.4	8.3		6.9	5.2	4.2
	60.1-70	11.6	8.7	6.9		5.8	4.3	3.5
	70.1-80	9.9	7.4	6.0		5.0	3.7	3.0
	80.1-90	8.7	6.5	5.2		4.3	3.3	2.6
	90.1-100	7.7	5.8	4.6		3.9	2.9	2.3
	100.1-110	6.9	5.2	4.2		3.5	2.6	2.1
	110.1-120	6.3	4.7	3.8		3.2	2.4	1.9
	>120	5.8	4.3	3.5		2.9	2.2	1.7
Current Smoker	40.1-50	10.9	8.1	6.5		5.4	4.1	3.3
	50.1-60	8.7	6.5	5.2		4.3	3.3	2.6
	60.1-70	7.2	5.4	4.3		3.6	2.7	2.2
	70.1-80	6.2	4.7	3.7		3.1	2.3	1.9
	80.1-90	5.4	4.1	3.3		2.7	2.0	1.6
	90.1-100	4.8	3.6	2.9		2.4	1.8	1.4
	100.1-110	4.3	3.3	2.6		2.2	1.5	1.2
	110.1-120	3.9	3.0	2.4		2.0	1.5	1.2
	>120	3.6	2.7	2.2		1.8	1.4	1.1

Confidence that low dose theophylline will be achieved using the above dosing strategy is provided from a detailed analysis of a COPD study that measured theophylline concentrations for 3 different theophylline dosing regimens[8]. In 33 COPD patients (mean weight (SD) weight of 64.6(14.3) Kg and age of 61.2(5.8) years) we found that the mean (SD) serum theophylline concentration at steady state when they received a mean of 252 (87) mg bd was 6.3 (2.1). This represents a clearance value of 51.6 ml/hr/kg. When their dose was increased to 430mg bd and then to 597(153) bd their mean (SD) steady state serum theophylline concentrations were 12.1(1.9) and 18.3(3.0) mg/L. This represents clearance values of 45.8ml/hr/kg and 42.1ml/hrkg. This will include smokers and non smokers (numbers of each not recorded) and the latter clearance value is similar to the 40ml/hr/kg used in the population pharmacokinetics modelling for the 'fast' category. Our other publications (n=83[9]; n=15[10] patients) on serum theophylline highlight our confidence of using low dose theophylline in this study.

References for Appendix 1

1. Hunt, S.N., Jusko, W.J. & Yurchak, A.M. Effect of smoking on theophylline disposition. Clin. Pharmac. Ther., 1976; 19: 546-51.
2. Powell, J.R., Thiercelin, J.F., Vozeh, S., Sansom, L. & Riegelman, S. The influence of cigarette smoking and sex on theophylline disposition. Am. Rev. Resp. Dis., 1977; 116: 17-23.
3. Jusko, W.J. Role of tobacco smoking in pharmacokinetics. J. Pharmacokinet. Biopharm.,1978; 6: 7-39.
4. Jusko. W.J., Schentag, J.J., Clark, J.H., Gardner, M. & Yurchak, A.M. Enhanced biotransformation of theophylline in marihuana and tobacco smokers. Clin. Pharmac. Ther., 1978; 24: 406-10.
5. Powell, J.R., Vozeh, S., Hopewell, P., Costello, J., Sheiner, L.B. & Riegelman, S. Theophylline disposition in acutely ill hospitalized patients. the effect of smoking, heart failure, severe airways obstruction and pneumonia. Am. Rev. Resp. Dis. 1978; 118: 229-38.
6. Hendeles L, Weinberger M & Bighley L. Disposition of theophylline after a single intravenous infusion of aminophylline. Am. Rev. Resp. Dis., 118, 97-103.
7. Cusack, J.G. Kelly, J. Lavan, J. Noel & O'Malley K. Theophylline kinetics in relation to age: the importance of smoking. Br. J. Clin. Pharmacol. 1980; 10: 109-14.
8. Chrystyn H., Mulley B.A. and Peake M.D. Dose response relation to oral theophylline in severe chronic obstructive airways disease. British Medical Journal, 1988, 297: 1506-10
9. Chrystyn H, Ellis JW, Mulley BA, Peake MD. The accuracy and stability of Bayesian theophylline predictions. Ther Drug Monit. 1988; 10: 299-305.
10. Chrystyn H, Mulley BA, Peake MD. The accuracy of a pharmacokinetic theophylline predictor using once daily dosing. Br J Clin Pharmacol 1987; 24: 301-7.
11. Chrystyn H, Ellis JW, Mulley BA, Peake MD.Bayesian derived predictions for twice daily theophylline under outpatient conditions and an assessment of optimal sampling times. Br J Clin Pharmacol. 1989; 27: 215-21.

APPENDIX 2 : SUMMARY OF PRODUCT CHARACTERISTICS FOR THEOPHYLLINE

From: http://www.medicines.org.uk/EMC/printfriendlydocument.aspx?documentid=1233
Downloaded on: 2 July 2014

Napp Pharmaceuticals Limited
Cambridge Science Park, Milton Road, Cambridge, Cambridgeshire, CB4 0GW
Telephone: +44 (0)1223 424 444
Fax: +44 (0)1223 424 441
WWW: http://www.napp.co.uk
Medical Information Fax: +44 (0)1223 424 912

Before you contact this company: often several companies will market medicines with the same active ingredient. Please check that this is the correct company before contacting them. Why?

Summary of Product Characteristics last updated on the eMC: 19/02/2014

Uniphyllin Continus tablets

1. Name of the medicinal product

UNIPHYLLIN® CONTINUS® 200 mg, 300 mg and 400 mg prolonged release tablets

2. Qualitative and quantitative composition

Tablets containing 200 mg, 300 mg or 400 mg of Theophylline Ph Eur.

For excipients, see 6.1.

3. Pharmaceutical form

Prolonged release tablet.

200 mg	Capsule shaped, white tablet, plain on one side and 'U200' on the other.
300 mg	Capsule shaped, white tablet, plain on one side and 'U300' on the other.
400 mg	Capsule shaped, white tablet with 'UNIPHYLLIN' on one side and 'U400' and the Napp logo on the reverse.

4. Clinical particulars

4.1 Therapeutic indications

For the treatment and prophylaxis of bronchospasm associated with asthma, chronic obstructive pulmonary disease and chronic bronchitis. Also indicated for the treatment of left ventricular and congestive cardiac failure.

Theophylline should not be used as first drug of choice in the treatment of asthma in children.

4.2 Posology and method of administration

Route of Administration

Oral.

The tablets should be swallowed whole and not crushed or chewed. Crushing or chewing the tablets may lead to a rapid release of theophylline with the potential for toxicity.

Patients vary in their response to xanthines and it may be necessary to titrate the dose on an individual basis.

The usual maintenance dose for adults and elderly patients is 200 mg 12 hourly. This may be titrated to either 300 mg or 400 mg dependent on the therapeutic response. Plasma theophylline concentrations should ideally be maintained between 5 and 15 mg/l. A plasma level of 5 mg/l probably represents the lower level of clinical effectiveness. Significant adverse reactions are usually seen at plasma theophylline levels greater than 20 mg/l. Patients may require monitoring of plasma theophylline levels when higher dosages are prescribed or when co-administered with medication that reduces theophylline clearance.

Children: The maintenance dose is 9 mg/kg twice daily. Some children with chronic asthma require and tolerate much higher doses (10-16 mg/kg twice daily). Lower dosages (based on usual adult dose) may be required for adolescents.

UNIPHYLLIN CONTINUS tablets should not be used in children below 6 years of age. Other dosage forms are available that are more suitable for children aged less than 6 years.

It may be appropriate to administer a larger evening or morning dose in some patients, in order to achieve optimum therapeutic effect when symptoms are most severe e.g. at the time of the 'morning dip' in lung function.

In patients whose night time or day time symptoms persist despite other therapy and who are not currently receiving theophylline, then the total daily requirement of UNIPHYLLIN CONTINUS tablets (as specified above) may be added to their treatment regimen as either a single evening or morning dose.

4.3 Contraindications

Porphyria; hypersensitivity to xanthines or any of the tablet constituents; concomitant administration with ephedrine in children.

Theophylline should not be administered to children under 6 months of age.

4.4 Special warnings and precautions for use

The patient's response to therapy should be carefully monitored – worsening of asthma symptoms requires medical attention.

Use with caution in patients with cardiac disease, peptic ulcer, hyperthyroidism, severe hypertension, hepatic dysfunction, chronic alcoholism or acute febrile illness.

Fever decreases the clearance of theophylline. It may be necessary to decrease the dose to avoid intoxication.

Caution should be exercised in elderly males with pre-existing partial outflow obstruction, such as prostatic enlargement, due to risk of urinary retention.

The half-life of theophylline may be prolonged in the elderly and in patients with heart failure, hepatic impairment or viral infections. Toxic accumulation may occur (see Section 4.9 Overdose). A reduction of dosage may be necessary in the elderly patient.

The hypokalaemia resulting from beta agonist therapy, steroids, diuretics and hypoxia may be potentiated by xanthines. Particular care is advised in patients suffering from severe asthma who require hospitalisation. It is recommended that serum potassium levels are monitored in such situations.

In case of insufficient effect of the recommended dose and in case of adverse events, theophylline plasma concentration should be monitored. Severe side effects (hypertonia, convulsions, supraventricular tachycardia) may indicate serum concentrations of theophylline above therapeutic levels. Serum concentrations should be checked urgently and a decrease in the dose of theophylline may be required.

Alternative treatment is advised for patients with a history of seizure activity.

It is not possible to ensure bioequivalence between different prolonged release theophylline products. Therefore patients, once titrated to an effective dose, should not be changed from one prolonged release theophylline preparation to a different prolonged release preparation without re-titration and clinical assessment.

4.5 Interaction with other medicinal products and other forms of interaction

The following increase clearance and it may therefore be necessary to increase dosage to ensure a therapeutic effect: aminoglutethimide, carbamazepine, isoprenaline, moracizine, phenytoin, rifampicin, ritonavir, sulphinpyrazone, barbiturates and hypericum perforatum. Plasma concentrations of theophylline can be reduced by concomitant use of the herbal remedy St John's Wort (hypericum perforatum). Smoking and alcohol consumption can also increase clearance of theophylline.

The following reduce clearance and a reduced dosage may therefore be necessary to avoid side-effects: allopurinol, carbimazole, cimetidine, ciprofloxacin, clarithromycin, diltiazem, disulfiram, erythromycin, fluconazole, interferon, isoniazid, methotrexate, mexiletine, nizatidine, norfloxacin, oxpentifylline, propafenone, propranolol, ofloxacin, thiabendazole, verapamil, viloxazine hydrochloride and oral contraceptives (see Section 4.9 Overdose). The concomitant use of theophylline and fluvoxamine should usually be avoided. Where this is not possible, patients should have their theophylline dose halved and plasma theophylline should be monitored closely.

Factors such as viral infections, liver disease and heart failure also reduce theophylline clearance (see Section 4.9 Overdose). There are conflicting reports concerning the potentiation of theophylline by influenza vaccine and physicians should be aware that interaction may occur. A reduction in dosage may be necessary in elderly patients. Thyroid disease or associated treatment may alter theophylline plasma levels. There is also a pharmacological interaction with adenosine, benzodiazepines, halothane, lomustine and lithium and these drugs should be used with caution.

Theophylline may decrease steady state phenytoin levels.

Xanthines can potentiate hypokalaemia resulting from $beta_2$ agonist therapy, steroids, diuretics and hypoxia. Particular caution is advised in severe asthma. It is recommended that serum potassium levels are monitored in such situations.

Co-administration with β-blockers may cause antagonism of bronchodilation; with ketamine may cause reduced convulsive threshold; with doxapram may cause increased CNS stimulation.

4.6 Pregnancy and lactation

There are no adequate data from well controlled studies of the use of theophylline in pregnant women. Theophylline has been reported to give rise to teratogenic effects in mice, rats and rabbits (See section 5.3). The potential risk for humans is unknown. Theophylline should not be administered during pregnancy unless clearly necessary. Theophylline is secreted in breast milk, and may be associated with irritability in the infant, therefore it should only be given to breast feeding women when the anticipated benefits outweigh the risk to the child.

4.7 Effects on ability to drive and use machines

No known effects.

4.8 Undesirable effects

The following adverse drug reactions have been reported in the post-marketing setting for theophylline. Frequencies of "not known" have been assigned as accurate frequencies cannot be estimated from the available clinical trial data.

**Immune system disorders**	Anaphylactic reaction
	Anaphylactoid reaction
	Hypersensitivity
**Metabolism and nutrition disorders**	Hyperuricaemia
**Psychiatric disorders**	Agitation
	Anxiety
	Insomnia
**Nervous system disorders**	Convulsions
	Dizziness
	Headache
	Tremor
**Cardiac disorders**	Atrial tachycardia
	Palpitations
	Sinus tachycardia
**Gastrointestinal disorders**	Abdominal pain
	Diarrhoea
	Gastric irritation
	Gastro-oesophageal reflux
	Nausea
	Vomiting
**Skin and subcutaneous tissue disorders**	Pruritus
	Rash
**Renal and urinary disorders**	Diuresis

	Urinary retention*

* Please refer to section 4.4 as theophylline may induce urinary retention in elderly males with pre-existing partial outflow obstruction.

4.9 Overdose

Over 3 g could be serious in an adult (40 mg/kg in a child). The fatal dose may be as little as 4.5 g in an adult (60 mg/kg in a child), but is generally higher.

Symptoms

Warning: Serious features may develop as long as 12 hours after overdosage with prolonged release formulations.

Alimentary features: Nausea, vomiting (which is often severe), epigastric pain and haematemesis. Consider pancreatitis if abdominal pain persists.

Neurological features: Restlessness, hypertonia, exaggerated limb reflexes and convulsions. Coma may develop in very severe cases.

Cardiovascular features: Sinus tachycardia is common. Ectopic beats and supraventricular and ventricular tachycardia may follow.

Metabolic features: Hypokalaemia due to shift of potassium from plasma into cells is common, can develop rapidly and may be severe. Hyperglycaemia, hypomagnesaemia and metabolic acidosis may also occur. Rhabdomyolysis may also occur.

Management

Activated charcoal or gastric lavage should be considered if a significant overdose has been ingested within 1-2 hours. Repeated doses of activated charcoal given by mouth can enhance theophylline elimination. Measure the plasma potassium concentration urgently, repeat frequently and correct hypokalaemia. BEWARE! If large amounts of potassium have been given, serious hyperkalaemia may develop during recovery. If plasma potassium is low, then the plasma magnesium concentration should be measured as soon as possible.

In the treatment of ventricular arrhythmias, proconvulsant antiarrhythmic agents such as lignocaine (lidocaine) should be avoided because of the risk of causing or exacerbating seizures.

Measure the plasma theophylline concentration regularly when severe poisoning is suspected, until concentrations are falling. Vomiting should be treated with an antiemetic such as metoclopramide or ondansetron.

Tachycardia with an adequate cardiac output is best left untreated. Beta-blockers may be given in extreme cases but not if the patient is asthmatic. Control isolated convulsions with intravenous diazepam. Exclude hypokalaemia as a cause.

5. Pharmacological properties

5.1 Pharmacodynamic properties

Theophylline is a bronchodilator. In addition it affects the function of a number of cells involved in the inflammatory processes associated with asthma and chronic obstructive airways disease. Of most importance may be enhanced suppressor, T-lymphocyte activity and reduction of eosinophil and neutrophil function. These actions may contribute to an anti-inflammatory prophylactic activity in asthma and chronic obstructive airways disease. Theophylline stimulates the myocardium and produces a diminution of venous pressure in congestive heart failure leading to marked increase in cardiac output.

5.2 Pharmacokinetic properties

Theophylline is well absorbed from UNIPHYLLIN CONTINUS tablets and at least 60% may be bound to plasma proteins.

An effective plasma concentration is considered to be 8-12 mg/l, although plasma concentrations up to 20 mg/l may be necessary to achieve efficacy in some cases. Do not exceed 20 mg/l.The main urinary metabolites are 1, 3-dimethyl uric acid and 3-methylxanthine. Theophylline is mainly excreted by the kidneys. About 10% is excreted unchanged.

5.3 Preclinical safety data

In studies in which mice, rats and rabbits were dosed during the period of organogenesis, theophylline produced teratogenic effects.

6. Pharmaceutical particulars

6.1 List of excipients

Hydroxyethylcellulose

Povidone (K25)

Cetostearyl Alcohol

Macrogol 6000

Talc

Magnesium Stearate

6.2 Incompatibilities

Not applicable.

6.3 Shelf life

Three years

6.4 Special precautions for storage

Do not store above 25°C.

6.5 Nature and contents of container

Blister packs consisting of aluminium foil sealed to 250 µm PVC with a PVdC coating of at least 40 gsm thickness, containing 56 tablets.

6.6 Special precautions for disposal and other handling

None.

7. Marketing authorisation holder

Napp Pharmaceuticals Ltd

Cambridge Science Park

Milton Road

Cambridge CB4 0GW

8. Marketing authorisation number(s)

PL 16950/0066-0068

9. Date of first authorisation/renewal of the authorisation

200 mg - 23 August 1979 / 15 May 2003

300 mg - 22 February 1988 / 15 May 2003

400 mg - 29 October 1982 / 15 May 2003

10. Date of revision of the text

14 November 2013

® UNIPHYLLIN, CONTINUS, NAPP and the Napp device (logo) are registered Trade Marks.

© 2010-2013 Napp Pharmaceuticals Ltd.

APPENDIX 3: PUBLICATION POLICY

AUTHORSHIP POLICY

1. *PRINCIPLES OF AUTHORSHIP*

The following principles of authorship have been derived from editorial publications from leading journals (see references) and are in accordance with the rules of the International Committee of Medical Journal Editors.

a. *Group authorship*

Group authorship will be appropriate for some publications, such as main reports. This will apply when the intellectual work underpinning a publication 'has been carried out by a group, and no one person can be identified as having substantially greater responsibility for its contents than others'. [1] In such cases the authorship will be presented by the collective title - The TWICS Trial Group - and the article should carry a footnote of the names of the people (and their institutions) represented by the corporate title. In some situations one or more authors may take responsibility for drafting the paper but all group members qualify as members; in this case, this should be recognised using the by-line 'Jane Doe and the Trial Group'.[2] Group authorship may also be appropriate for publications where one or more authors take responsibility for a group, in which case the other group members are not authors but may be listed in the acknowledgement (the by-line would read 'Jane Doe for the Trial Group'). [2]

b. *Individual authorship*

Other papers, such as describing satellite studies, will have individual authorship. In order to qualify for authorship an individual must fulfil the following criteria[1]:

i. each author should have participated sufficiently in the work represented by the article to take public responsibility for the content.
ii. participation must include three steps:
   - conception or design of the work represented by the article OR analysis and interpretation of the data OR both; AND
   - drafting the article or revising it for critically important content; AND
   - final approval of the version to be published.

Participation solely in the collection of data is insufficient by itself and those persons who have contributed intellectually to the article but those contributors do not justify authorship may be acknowledged and their contribution described.[1]

c. *Determining authorship*

Tentative decisions on authorship should be made as soon as possible.[1] These should be justified to, and agreed by, the Project Management Group. Any difficulties or disagreements will be resolved by the Steering Committee.

2. *AUTHORSHIP FOR PUBLICATION ARISING FROM TWICS*

a. *Operationalising authorship rules*

We envisage two types of report (including conference presentations) arising from the TWICS trial and its associated projects:

i. Reports *of work arising from the main TWICS trial*

If all grant-holders and research staff fulfil authorship rules, group authorship should be used under the collective title of 'The TWICS Trial Group'; if one or more individuals have made a significant contribution above and beyond other group members but where all group members fulfil authorship rules, authorship will be attributed to 'Jane Doe and the TWICS Trial Group'.

ii. Reports *of satellite studies and subsidiary projects*
Authorship should be guided by the authorship rules outlined in Section 1 above. Grant-holders and research staff not directly associated with the specific project should only be included as authors if they fulfil the authorship rules. Grant-holders and research staff who have made a contribution to the project but do not fulfil authorship rules should be recognised in the Acknowledgement section. The role of the TWICS Trial Group in the development and support of the project should be recognised in the Acknowledgement section. The lead researcher should be responsible for ratifying authorship with the Project Management Group.

For reports which specifically arise from the TWICS trial but where all members do not fulfil authorship rules (for example, specialist sub-study publications), authorship should be attributed to 'Jane Doe for the TWICS Trial Group'. If individual members of the group are dissatisfied by a decision, they can appeal to the Management Group for reconciliation. If this cannot be achieved, the matter should be referred to the Steering Group.

b. Quality assurance
Ensuring quality assurance is essential to the good name of the trial group. For reports of individual projects, internal peer review among members of the Project Management Group is a requirement prior to submission of papers. All reports of work arising from the TWICS trial including conference abstracts should be peer reviewed by the Project Management Group.

The internal peer review for reports of work arising from the TWICS project is mandatory and submission may be delayed or vetoed if there are serious concerns about the scientific quality of the report. The Project Management Group will be responsible for decisions about submission following internal peer review. If individual members of the group are dissatisfied by decisions, the matter may be referred to the Steering Group.

The Project Management Group undertake to respond to submission of articles for peer review at the Project Management Group Meeting following submission (assuming the report is submitted to the trial secretariat in Aberdeen at least two weeks prior to the meeting).

References for Appendix 3

1. Huth EJ (1986). Guidelines on authorship of medical papers. Annals of Internal Medicine, 104, 269-74.
2. Glass RM (1992). New information for authors and readers. Group authorship, acknowledgements and rejected manuscripts. Journal of the American Medical Association, 268, 99.
